# Phonon engineering of atomic-scale defects in superconducting quantum circuits

**DOI:** 10.1126/sciadv.ado6240

**Published:** 2024-09-13

**Authors:** Mo Chen, John Clai Owens, Harald Putterman, Max Schäfer, Oskar Painter

**Affiliations:** ^1^ Thomas J. Watson, Sr., Laboratory of Applied Physics, California Institute of Technology, Pasadena, CA 91125, USA.; ^2^ Institute for Quantum Information and Matter, California Institute of Technology, Pasadena, CA 91125, USA.; ^3^ Kavli Nanoscience Institute, California Institute of Technology, Pasadena, CA 91125, USA.; ^4^ AWS Center for Quantum Computing, Pasadena, CA 91125, USA.

## Abstract

Noise within solid-state systems at low temperatures can typically be traced back to material defects. In amorphous materials, these defects are broadly described by the tunneling two-level systems (TLSs) model. TLS have recently taken on further relevance in quantum computing because they dominate the coherence limit of superconducting quantum circuits. Efforts to mitigate TLS impacts have thus far focused on circuit design, material selection, and surface treatments. Our work takes an approach that directly modifies TLS properties. This is achieved by creating an acoustic bandgap that suppresses all microwave-frequency phonons around the operating frequency of a transmon qubit. For embedded TLS strongly coupled to the transmon qubit, we measure a pronounced increase in relaxation time by two orders of magnitude, with the longest *T*_1_ time exceeding 5 milliseconds. Our work opens avenues for studying the physics of highly coherent TLS and methods for mitigating noise within solid-state quantum devices.

## Teaser

Nanoscale engineering of materials may pave the way to mitigate noise in solid-state quantum devices.

## INTRODUCTION

Glassy materials exhibit abnormal thermal transport behaviors at low temperatures, *T* < 1 K. These anomalies include specific heat and thermal conductivity that deviate from predictions of the Debye model. This is counter-intuitive because the wavelengths of relevant phonons at low temperatures are too long to distinguish between structurally amorphous and crystalline solids. To address this mystery, Phillips (*1*) and Anderson *et al.* (*2*) independently proposed the ubiquitous existence of microscopic two-level systems (TLSs), which are defect states tunneling between two nearly equivalent local potential wells. These TLS defects are known to distribute nearly uniformly over a broad frequency range, and they have both elastic and electric dipoles that allow them to couple to strain and electric fields (*3*). The TLS model successfully explains the aforementioned thermal anomalies of glassy materials, as well as their acoustic and dielectric behaviors at low temperatures. In addition, because of the omnipresence of TLS in amorphous materials, their wide frequency distribution, and their ability to couple through both phonons and photons, TLS have been associated with noise in various solid-state quantum systems, including superconducting (SC) quantum circuits (*4*–*8*), nanomechanical resonators (*9*–*11*), and optomechanical cavities (*12*, *13*).

In the context of SC quantum circuits, TLS have been identified as a primary limitation to the energy lifetime, coherence, and overall stability of the physical qubits being explored for scalable quantum computing architectures (*3*, *5*–*8*, *14*–*19*). TLS are thought to reside primarily at the amorphous material interfaces that make up the physical qubit device and cause dielectric loss through the interaction between their electric dipoles and the electric field of the qubit. The interaction leads to a two-step energy dissipation process, where energy first transfers from the SC qubit to resonant TLS and subsequently dissipates into the local environment of the TLS (*20*–*23*). Despite awareness of the two-step dissipation process for SC qubit decay, past research efforts have focused on investigating and mitigating the first step, namely, the energy decay from the SC qubit to TLS. This choice is in part because of the challenge in accessing and controlling atomic-scale TLS defects (*24*, *25*) and therefore in one’s ability to modify the second step of the dissipation process. As a result, TLSs have long been viewed as an intrinsic material defect to be avoided (*14*, *19*, *26*, *27*). In the pursuit of better SC qubits, material investigations have focused on finding superconductors with a surface oxide layer that has low TLS density (*28*–*30*). Similarly, circuit designs of SC qubits aim to minimize the electric-field strength of the electromagnetic field produced by the qubit at material interfaces to reduce the interaction between the SC qubit and TLS (*14*, *26*, *27*). These efforts have led to microwave-frequency SC qubits with energy relaxation times that extend over hundreds of microseconds (*18*).

**Fig. 1.**
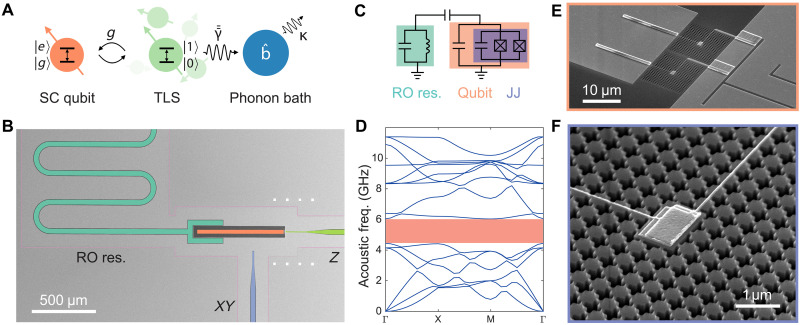
A hybrid platform for phonon engineering in superconducting quantum circuits. (**A**) Schematic of the two-step energy dissipation process in a SC qubit. Energy first decays from the SC qubit to a bath of near-resonant TLS, with coupling rate *g*, and then dissipates into the local environment of TLS. This environment is presumed to be dominated by a bath of phonon modes $\hat{b}$ , relaxing at a rate κ, and interacting with the elastic dipole $\bar{\bar{\gamma }}$ of the TLS. (**B**) SEM image (false colored) of the fabricated hybrid transmon qubit device. Each qubit couples to its dedicated λ/4 readout (RO) resonator (turquoise), *Z* control line (green) and *XY* control line (blue). The entire device, outlined in pink, is suspended on the 220-nm-thick Si device layer, which is released from the underlying 3-μm-thick oxide BOX layer of the SOI chip. (**C**) Circuit diagram of the transmon qubit and readout resonator. Approximately 40% of the transmon capacitance comes from the shunt capacitor (orange), and 60% from the JJs. (**D**) Simulated acoustic band structure of the Si cross-shield unit cell, with the acoustic bandgap centered around 5.1 GHz shaded in pink. (**E**) Zoomed-in view of the SQUID loop of the transmon qubit device. The SQUID loop is formed with two JJs in parallel between the shunt capacitor and ground. Each JJ is fabricated on top of a micron-scale Si platform tethered to the SOI substrate by a cross-shield acoustic bandgap structure. (**F**) Detailed SEM image of one of the JJs, showing the cross-shield patterning of the acoustic bandgap structure. The contacts from the shunt capacitor and ground to the top and bottom electrodes of the JJ, respectively, are visible as narrow Al leads that run across the connected cross-shield lattice.

In the hopes of further understanding TLS and improving SC qubits, in this work, we take direct aim at modifying the second step of the dissipation process of SC qubits, namely, the interaction of TLS with the reservoir of phonons of the material host as illustrated in Fig. 1A. The phonon bath is targeted due to the roughly five-orders-of-magnitude difference in the speed of sound and the speed of light in materials, and the correspondingly much larger density of states (DOS) at microwave frequencies for phonons versus photons. For typical Debye-level electric dipole moments and eV-level deformation potentials of TLS, this makes the dominant bath that of phonons. We design and fabricate a frequency-tunable transmon qubit (*31*, *32*) with its Josephson junctions (JJs) embedded in an engineered acoustic structure that features a GHz-wide acoustic bandgap, as shown in Fig. 1 (B to F). TLS within the junctions and with transition frequency within the acoustic bandgap range, experience a suppressed two-dimensional (2D) phonon DOS, which effectively shields them from resonant decay into the phonon bath. Using the transmon qubit as a quantum sensor, we are able to individually address and characterize the coherence properties of strongly coupled TLS within the JJs of the electric qubit. Our experimental results show that the *T*_1_ times for TLS with resonant frequency lying inside the acoustic bandgap are, on average, extended by two orders of magnitude compared to when the TLS frequency lies outside the acoustic bandgap. The coherence and the temperature dependence of the *T*_1_ relaxation process of the acoustically shielded TLS are also studied, indicating that coherence is limited to the microsecond level due to low-frequency noise, and thermally activated relaxation channels open up above 75 mK.

## RESULTS

### Transmon qubit with acoustically shielded junctions: Design and fabrication

We fabricate our transmon qubit device on a silicon-on-insulator (SOI) substrate (*33*), which allows for nanoscale fabrication of high-quality acoustic bandgap structures in the microwave frequency range (see the “Methods” section in the Supplementary Materials for design and fabrication details). Figure 1 (B, E, and F) shows scanning electron micrographs (SEM) of various parts of the device. The transmon qubit has a shunt capacitor, which is used to couple to a coplanar-waveguide resonator for readout of the qubit state. It also has two aluminum-aluminum oxide-aluminum (Al-AlO*_x_*-Al) JJs forming a magnetic flux sensitive SQUID loop for tuning of the electric qubit state via a current-carrying *Z* control line. An *XY* control line is added for direct charge excitation of the qubit. The transmon qubit is unremarkable in its design, except for the fact that each JJ in the SQUID loop is located on top of a suspended Si platform formed by the release of the 220-nm-thick Si device layer from the SOI substrate and tethered to the SOI substrate by nine periods of an acoustic bandgap structure (Fig. 1, E and F). The acoustic bandgap structure of this work is based on a cross-shield design (*13*, *34*), which has a 1.372-GHz-wide acoustic bandgap centered at 5.128 GHz (see the “Acoustic bandgap metamaterials” section in the Supplementary Materials). In theory, this effectively isolates the JJs of the transmon qubit, and any TLS defects that may be within the amorphous oxide layer of each junction, from acoustic modes of the SOI substrate (see the “Localized acoustic phonon modes in the bandgap” section in the Supplementary Materials for a discussion on localized acoustic phonon modes).

The TLS within the acoustically isolated JJs are distinguished from other TLS in different regions of the circuit by their signature strong coupling to the transmon qubit due to the strong electric field of the qubit mode in the atomically thin AlO*_x_* barrier layer of the JJs. To increase the occurrence of these TLS of interest, the JJs of our device are chosen to have a relatively large area of 0.83 μm^2^ each. The AlO*_x_* barrier layer is also grown slightly thicker to keep the junction energy *E*_J_ uninfluenced by the change in junction size (see the “Device fabrication” section in the Supplementary Materials for details). As a result, the JJs have a substantial junction capacitance in addition to the nonlinear inductance, which is similar to the merged-element transmon qubit (*35*, *36*). In the current design, the JJs account for approximately 60 fF or 60% of the total transmon capacitance. The remaining qubit capacitance comes from an additional planar shunt capacitor, which is varied for each transmon qubit. This variation in shunt capacitance produces a series of qubit devices with upper sweet-spot frequency covering a range of 5.44 to 6.48 GHz, facilitating the characterization of the entire acoustic bandgap of the designed acoustic shield.

### Transmon qubit and TLS characterization

Characterization of transmon qubit devices, and any coupled TLS, is performed in a dilution refrigerator, where the chip-scale sample containing the devices is mounted to the mixing chamber plate of the fridge. The fridge reaches a base temperature of 7 mK, which cools down both the SC qubit and TLS close to their respective ground states. The transmon qubit is first characterized in the time domain with pulsed excitation and dispersive readout. The transmon qubits of this work are measured to have excited-to-ground state relaxation times of *T*_1_ ∼ 3 μs, which is comparable to the best reported SOI qubit (*33*). We attribute the qubit *T*_1_ relaxation to both dielectric loss at the shunt capacitor and Purcell decay to the readout resonator. Further details of the qubit characterization and the qubit parameters for all seven qubit devices studied in this work can be found in the “Device design” section in the Supplementary Materials.

**Fig. 2.**
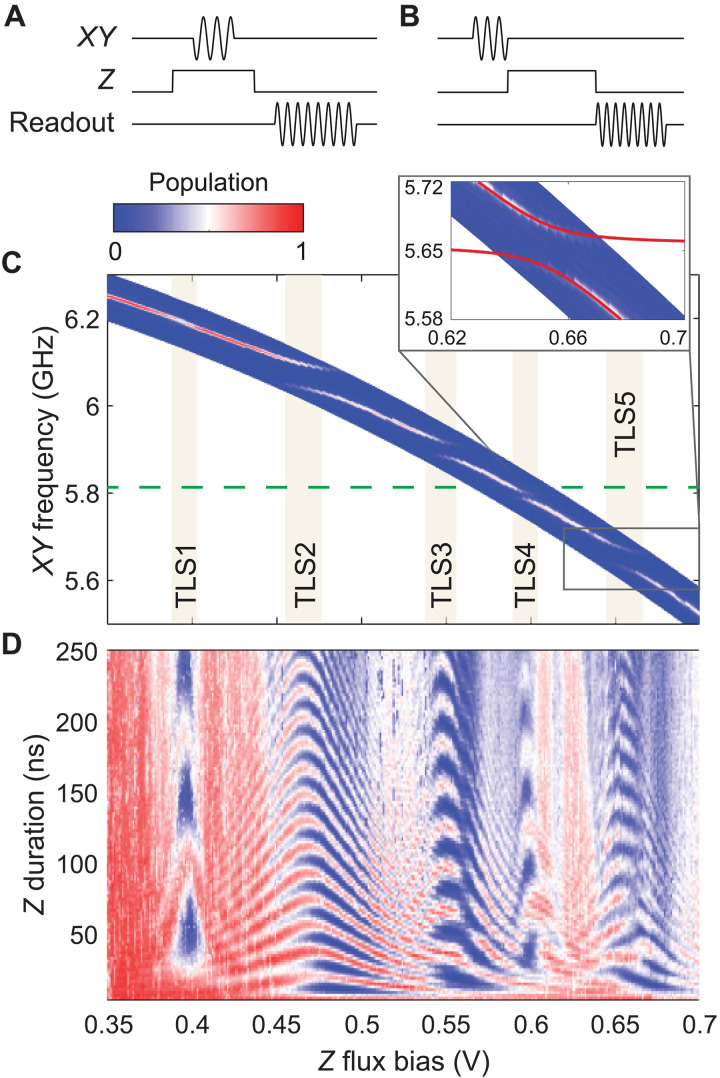
Characterization of a hybrid transmon-TLS system. (**A**) Pulse sequence used for microwave spectroscopy of the qubit and (**C**) corresponding measured transmon qubit spectrum for *Q*_1_ of Chip-A. In this measurement protocol, a *Z*-pulse flux biases the transmon qubit away from its flux-insensitive sweet spot. An overlapping *XY* pulse of 100 ns duration probes the excitation of the transmon qubit at different flux biases. When the transmon is in resonance with a TLS, their hybridization results in avoided crossings. For *Q*_1_ of Chip-A, we measure five distinct TLS, labeled TLS1 to TLS5. Notably, the avoided crossings of TLS4 and TLS5 are situated within the simulated acoustic bandgap, with the upper frequency bandedge indicated by the green dashed line. The inset provides a magnified view of the avoided crossing of TLS5. The red solid line in the inset is a fitting curve with ω_TLS5_/2π = 5.6563 GHz and *g*/2π = 21.7 MHz. (**B**) Pulse sequence of the transmon-TLS SWAP gate spectroscopy and (**D**) corresponding measured transmon-TLS SWAP spectrum for device *Q*_1_ of Chip-A. In this measurement protocol, the transmon is first excited by an *XY* π-pulse and then tuned by a *Z* pulse with varying amplitude and duration, and lastly the transmon qubit population is dispersively read out upon tuning back to its starting frequency. The resulting chevron patterns correspond to vacuum Rabi oscillations between the transmon qubit and the strongly coupled TLS1 to TLS5.

Once characterized, the transmon qubit is used as a quantum sensor to identify individual TLS in the JJs that experience a structured acoustic environment. Using the pulse sequence depicted in Fig. 2A, we perform pulsed microwave spectroscopy to explore the electrically-active transitions of a given transmon qubit device. The measured microwave spectrum of one such transmon qubit device (Chip-A, Q_1_) is shown in Fig. 2C, with qubit frequency tuned between 5.5 and 6.3 GHz using a flux bias pulse via the *Z* control line. Strong couplings of the transmon to five TLS (labeled TLS1 to TLS5) manifest as avoided crossings in the spectrum, from which we extract the TLS frequencies and coupling strengths *g* to the transmon qubit. In these experiments, the microwave power on the *XY* control line is chosen to yield approximately 100-ns transmon π-pulses, which determines the power-broadened qubit linewidth and allows us to resolve TLS with a coupling strength of *g* ≳ 5 MHz. The strong couplings of TLS1 to TLS5 are a signature that these TLS are physically located inside the JJs of Q_1_ on Chip-A and, hence, inside the acoustic bandgap structures.

Next, to probe further the properties of the strongly coupled TLS, we calibrate coherent SWAP operations between transmon qubit and TLS states using SWAP spectroscopy (*37*, *38*), as illustrated in Fig. 2B. Figure 2D shows a representative measurement of SWAP spectroscopy performed on device Q_1_ of Chip-A, where five pronounced vacuum Rabi oscillation patterns appear at the same pulsed flux-bias amplitudes as the previously measured anticrossings for TLS1 to TLS5. This confirms that these fringes arise from the resonant exchange interactions between the transmon qubit and individual strongly coupled TLS and signifies the coherent nature of these TLS. On the basis of these vacuum Rabi oscillations, we identify optimal SWAP gates for each TLS.

Using the SWAP gate, we are able to selectively prepare any one of the five TLS in their first excited state. Moreover, sequential application of this technique allows us to look for the presence of second (and higher-order) excited states of the TLS. The absence of observable higher-order excited states (see the “Anharmonicity of TLS” section in the Supplementary Materials) indicates that the strongly coupled TLS to the transmon qubits measured here are highly anharmonic. This eliminates any concerns that the TLS-like behavior observed in this study originates from high-*Q* harmonic acoustic modes of the acoustic bandgap structure.

### T1 lifetime of acoustically shielded TLS

**Fig. 3.**
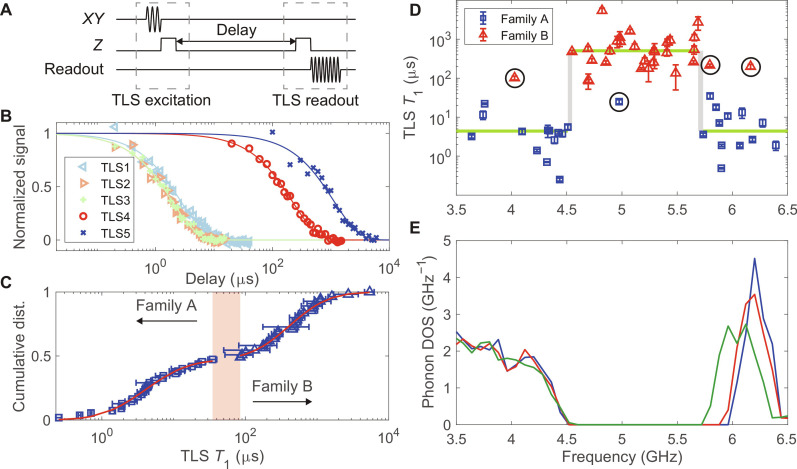
TLS *T*_1_ relaxations in an engineered acoustic bath. (**A**) Pulse sequence to measure the *T*_1_ energy relaxation time of TLS. The qubit is first driven to its excited state via an *XY* π-pulse. Then the excitation is transferred to the TLS using a *Z* SWAP pulse. After a variable delay, the TLS state is swapped back to the transmon qubit and the qubit population is subsequently read out. (**B**) Relaxation curves for TLS1 to TLS5 of *Q*_1_ on Chip-A, identified in the microwave spectroscopy of Fig. 2. The measured signals are normalized and fitted to a decaying exponential. (**C**) Cumulative distribution of TLS *T*_1_ relaxation times from 55 TLSs measured across two chips, seven devices, and two cooldowns. A gap between 35 and 85 μs (pink shade) divides the cumulative distribution into families A and B. Each family is fitted to a log-normal distribution (red lines). (**D**) TLS *T*_1_ relaxation times plotted against their frequencies. TLS in family A (blue squares) and family B (red triangles) exhibit strong correlation with TLS frequency. The gray shaded regions represent the edges of a central frequency band where almost all of the TLSs in family B reside, and outside of which almost all of the TLSs in family A reside. The green line is a guide for the eye, representing median *T*_1_ values for TLS with transition frequency inside and outside the central frequency region. Four outlier TLS from families A and B are marked by black circles. (**E**) Simulated phonon DOS of an infinitely-periodic cross-shield acoustic bandgap structure. Blue, red, and green curves represent slightly different acoustic structure unit cells due to variations in the Al leads from the JJs (see the “Device design” section in the Supplementary Materials).

To characterize the lifetime of TLS, we begin by preparing a TLS in its excited state using the SWAP gate and then let the TLS relax for a variable amount of time. Last, we map the TLS state back to the transmon using a second SWAP gate and measure the final transmon state using its dispersive readout circuit (Fig. 3A). During the TLS relaxation time, the transmon qubit is typically tuned to its uppermost frequency, far from resonance with the TLS to avoid Purcell decay of the TLS through the transmon qubit. The resulting *T*_1_ relaxation curves for the five characterized TLS of Q_1_ on Chip-A are shown in Fig. 3B, with TLS1 to TLS3 having *T*_1_ ∼ 2 μs, while TLS4 and TLS5 exhibit two to three orders-of-magnitude longer relaxation times of *T*_1,TLS4_ = 215 ± 15 μs and *T*_1,TLS5_ = 1100 ± 200 μs, respectively (here the one SD uncertainty in the *T*_1_ is quoted). Notably, both TLS4 and TLS5 have transition frequencies that lie within the expected acoustic bandgap of the crosss-shield structure based on numerical finite-element simulations, whereas TLS1 to TLS3 all have frequencies above the simulated bandgap.

To gather further statistical data on the correlation between the *T*_1_ of strongly coupled TLS and their transition frequency, we examined TLS across seven transmon qubit devices, on two different fabricated chips. In addition, we thermally cycled the devices up to room temperature and back down to milliKelvin temperatures to redistribute the TLS frequencies (*39*). In total, 56 TLSs (or more accurately TLS with unique frequencies) have been characterized. These TLSs span frequencies from 3.7421 to 6.3935 GHz, and their *T*_1_ values range from 0.25 ± 0.02 μs to 5400 ± 800 μs. For a full list of measured TLS parameters, see the “TLS parameters” section in the Supplementary Materials. Here, we focus on the statistical properties of the TLS and show compelling evidence that the extended TLS *T*_1_ relaxation times, as observed in TLS4 and TLS5, originate from the acoustic bandgap.

We begin our analysis by plotting in Fig. 3C the cumulative distribution of TLS versus their measured *T*_1_ relaxation time (here, we have excluded one TLS data point whose *T*_1_ value is in proximity to the Purcell limit set by the transmon qubit; see the “TLS parameters” section in the Supplementary Materials). From this plot, we identify a gap in measured *T*_1_ times, between 35 and 85 μs (pink shaded region). This gap in *T*_1_ divides the TLS naturally into two distinct families, referred to as family A (blue squares) and family B (blue triangles). Each family is fitted to a log-normal distribution, represented by the red solid lines. The fitted parameters yield median *T*_1_ values of 4.1 ± 0.2 μs and 414 ± 17 μs for family A and B, respectively (we use the median as opposed to the mean due to the large skewness of 8.3 and 6.2 for the TLS *T*_1_ distributions of the two families).

Next, we plot in Fig. 3D the measured TLS *T*_1_ relaxation times against their frequencies, marking those TLS in family A with blue squares and those in family B with red triangles. As is clearly visible, the two TLS families, categorized solely by their distinct *T*_1_ values, exhibit a strong correlation with frequency. Specifically, TLS in family A predominantly occupy frequencies outside a frequency band centered around 5.1 GHz, whereas those in family B mostly reside within this frequency band. We can define this central frequency band quantitatively by using the following cost function

$$𝒞\left(f_{1},f_{2}\right)=\text{log}\left[1-F_{A}\left(f_{1},f_{2}\right)\times F_{B}\left(f_{1},f_{2}\right)\right]$$ (1)

where the central frequency band is defined between lower and upper bandedges *f*_1_ and *f*_2_, respectively. *F*_A_(*f*_1_, *f*_2_) denotes the fraction of TLS in family A whose frequencies lie outside this frequency band, while *F*_B_(*f*_1_, *f*_2_) represents the fraction of TLS in family B that fall within this frequency band. Upon minimizing the cost function, we obtain 𝒞_min_ = −1.98, with the lower bandedge *f*_1_ lying between 4.510 and 4.547 GHz, and the upper bandedge *f*_2_ lying between 5.690 and 5.735 GHz. These empirically defined bandedges are marked as vertical gray shaded regions in Fig. 3D. This can be compared to the expected frequency bandgap region of the cross-shield acoustic structure that the JJs of the transmon qubits are embedded within. In Fig. 3E, we present the numerically simulated phonon DOS for three slightly different unit cells of the acoustic cross-shield, taking into account variations in the Al leads that connect the JJs to the rest of the circuit (see the “Device design” and the “Phonon density of states” sections in the Supplementary Materials). Even for the simulation with the heaviest loading by the Al leads (green curve in Fig. 3E), we find excellent correspondence between the frequency band defined by high TLS *T*_1_ values and the acoustic bandgap with zero phonon DOS of the simulated cross-shield structure.

The above correlations between TLS *T*_1_, TLS frequency, and the designed acoustic bandgap frequency serve as compelling evidence that the observed several orders-of-magnitude increase in TLS *T*_1_ relaxation time, from a median of *M*_out,2D_(*T*_1_) = 4.4 μs outside the central frequency band region to a median of *M*_in,2D_(*T*_1_) = 505 μs inside the central frequency band region, originates from the presence of an acoustic bandgap in the acoustic bath seen by the strongly coupled TLS within the JJs. This result also indicates that for TLS that couples to the electric field of microwave-frequency SC qubits, the dominant relaxation channel is spontaneous phonon emission into the acoustic bath, in agreement with the two-step dissipation chain shown in Fig. 1A. There are, however, several outliers in the measured TLS data. These are marked by black circles in Fig. 3D. We attribute two outliers, the TLS from family A that has low *T*_1_ but lies well inside the average phononic bandgap near 5 GHz and the TLS of family B with high *T*_1_ lying just outside the central frequency band at 5.8 GHz, to device-to-device alterations in the bandgap associated with the fabrication precision in our electron beam lithography and Si etching processes (see discussion in the “Acoustically shielded TLS on individual devices” section in the Supplementary Materials). Despite these outliers, the consistency of the inferred bandgap region from all seven devices indicates that the fabrication process, although not perfect, is relatively accurate on the scale of a percent SD.

The two TLSs of family B with high *T*_1_ values that are far from the central frequency band, at approximately 4 and 6.2 GHz, represent a different type of outlier (*40*). We believe that these TLS, although lying outside the cross-shield bandgap, have either acoustic dipole orientations that are orthogonal to the polarization of the acoustic modes of the cross-shield structure or are decoupled from the acoustic bulk phonon modes of the SOI substrate due to the finite extent of the cross-shield and acoustic reflections at its perimeter. These effects might also explain a portion of the large variation seen in the *T*_1_ values for TLS outside of the acoustic bandgap, although varying acoustic dipole strength between TLS would also contribute to the observed *T*_1_ variance. This discussion draws attention to the fact that all of the measured TLSs in this work live within a 2D Si membrane, with an effective 2D phonon DOS. Comparing our results to previous studies of TLS in the JJs of a phase qubit fabricated on a high resistivity Si substrate, where the median *T*_1_ values were of order *M*_3D_(*T*_1_) ∼ 200 ns (*39*), highlights that the observed increase in TLS *T*_1_ within the acoustic bandgap of our structures is three orders of magnitude above that for TLS in a 3D bulk material.

### TLS coherence and temperature dependence of *T*_1_ relaxation

The significantly extended TLS *T*_1_ time naturally raises questions about their coherence time. There is also the question of what limits the TLS *T*_1_ values once the direct resonant coupling to a phonon bath is removed. Here, we perform further studies on TLS5 of device *Q*_1_ on Chip-A, which displays a long *T*_1_ = 1100 ± 200 μs, making it a sensitive probe of these effects. Coherent control of the TLS can be achieved by sending a strong microwave pulse resonant with the TLS down the *XY* control line of the transmon qubit (*41*). This pulse is able to directly control the TLS due to the hybridization between the transmon qubit and the TLS, as described in the “Direct control of TLS” section in the Supplementary Materials. We calibrate control pulses for TLS5 of Q_1_ on Chip-A and perform Ramsey spectroscopy to characterize its $T_{2}^{*}$.

**Fig. 4.**
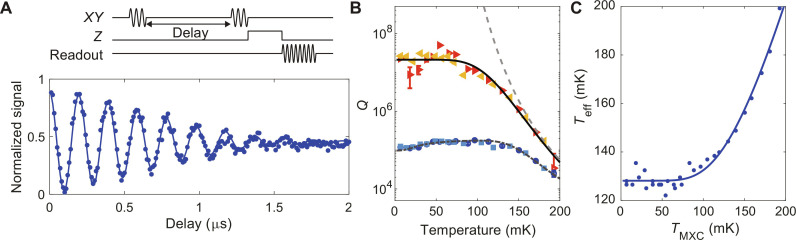
Coherence and temperature-dependent *T*_1_ relaxation of TLS5. (**A**) Top: Pulse sequence to measure the TLS Ramsey spectroscopy. The sequence consists of two TLS π/2-pulses separated by a variable delay, followed by a TLS-transmon SWAP gate and subsequent transmon readout. Bottom: Measured Ramsey curve for TLS5. Fitting the data to an oscillatory decaying curve yields $T_{2}^{*}=0.91\pm 0.05\;\mu s$. Blue dot markers represent experimental data, and the blue solid line represents the fit to the data. The *XY* control line driving power for the TLS is 17 dB stronger than what is typically used for controlling the transmon qubit. This increased driving power allows for fast TLS control with *t*_π_ = 160 ns. (**B**) Plot of the *Q* factor of both TLS5 and the transmon qubit as a function of the mixing chamber plate temperature. The red (dark blue) markers denote measurements of the TLS (transmon qubit) during device warm-up (WU), and the yellow (light blue) markers during the device cooldown (CD). The gray dashed line corresponds to a phenomenological model of QP damping of TLS using the mixing chamber plate temperature. The black solid line represents a correction to the gray dashed line, when using the effective temperature from (C). Gray dash-dotted line is a fit to the transmon curve with a model including thermal saturation of weakly coupled TLS and damping from thermally-activated QPs. (**C**) Plot of the effective temperature against the mixing chamber plate temperature. The effective temperature is deduced by assuming a single TLS energy relaxation channel of QPs. The empirical fit assumes the functional form $T_{\text{eff}}\left(T_{\text{MXC}}\right)=A\sqrt{1+B\text{tanh}\left(C/T_{\text{MXC}}\right)}/\text{tanh}\left(C/T_{\text{MXC}}\right)$.

Applying the Ramsey sequence to TLS, in Fig. 4A, we plot the excited state population of the transmon qubit mapped from the TLS as a function of the free precession time of the TLS. Fitting the resulting pattern to an oscillatory decaying curve, $A\text{cos}\left(\omega t+\phi_{0}\right)\text{exp}\left[-{\left(t/T_{2}^{*}\right)}^{n}\right]+B$ , yields $T_{2}^{*}=0.91\pm 0.05\;\mu s$, with *n* = 1.8 ± 0.3. The obtained TLS coherence time does not show a significant improvement over TLS without the engineered acoustic environment (*38*, *41*). This is perhaps to be expected, as the decoherence rate of TLS, Γ_2_ = Γ_1_/2 + Γ_ϕ_ has both an energy lifetime component (Γ_1_ = 1/*T*_1_) and a pure-dephasing component (Γ_ϕ_). The dephasing rate Γ_ϕ_ is thought to be dominated by frequency noise from the fluctuations of a bath of thermally activated TLS with frequencies *ℏ*ω ≲ *k*_B_*T* (*42*). This model is consistent with the exponent of the TLS coherence decay: Being close to quadratic, it is associated with coherent-like, low-frequency noise on the TLS transition frequency. Although the microwave-frequency acoustic bandgap significantly reduces Γ_1_ = 1/*T*_1_, the properties of these low-frequency TLS and their associated Γ_ϕ_ contribution are not expected to be fundamentally altered by our acoustic structuring. However, this low-frequency noise can be effectively filtered out through techniques such as dynamical decoupling, allowing TLS *T*_2_ to approach twice its extended *T*_1_ time. This technique has been extensively applied to improve the coherence time of electronic spin qubits subject to a noisy low-frequency nuclear spin bath (*43*) and will be the subject of future work with TLS in our acoustically engineered devices.

Last, in Fig. 4B, we present the temperature dependence of the *T*_1_ relaxation rate of TLS5 as we warm up the mixing chamber plate of the fridge from a base temperature of 7 to 193 mK and cool down back to base temperature. No hysteresis is observed between the warm-up (red markers) and cooldown (yellow markers) paths. Here, we plot the quality factor, defined as *Q* = ω*T*_1_, to compare the relaxation of both TLS5 at transition frequency ω_TLS5_/2π = 5.65 GHz and the transmon qubit at transition frequency ω_q_/2π = 6.48 GHz. The variation of the transmon *Q* factor with temperature agrees with a widely adopted model that considers effects from thermal saturation of weakly coupled resonant TLS and the thermal excitation of quasiparticles (QPs) in the SC Al layers of the transmon qubit (*4*, *44*, *45*). A fit of this model (see the “Dependence of relaxation on temperature” section in the Supplementary Materials for details) to the transmon data is shown as a gray dash-dotted line.

The *Q* factor of the TLS, on the other hand, stays roughly constant at *Q* ∼ 2.5 × 10^7^ for temperatures below 75 mK and then drops by three orders of magnitude to *Q* < 5 × 10^4^ at 193 mK. For temperatures above 150 mK, the drop in TLS *Q* factor looks to follow that of the transmon qubit. Fitting a similar QP loss model to the TLS data in this region yields the dashed curved in Fig. 4B. As can be seen, the plateau in the TLS *Q* factor at the lowest temperatures is not captured by this simple model. Possible explanations for the limited TLS *Q* factor below 75 mK include temperature-independent phenomena such as TLS coupling to heavily damped grain-boundary motion in the polycrystalline Al films (*10*, *46*) or coupling to nonequilibrium QPs induced by high-energy particle events (*47*). Using the high temperature fit of the QP model to the TLS *Q* factor data and then fitting for an effective temperature *T*_eff_ for the QPs that tracks the measured TLS *Q*-factor data at lower mixing plate temperatures (*T*_MXC_) yield the temperature curve shown in Fig. 4C. The corresponding fitting curve for the TLS *Q* factor assuming *T*_eff_(*T*_MXC_) is shown as a solid black line in Fig. 4B (the transmon fit using *T*_eff_ remains largely unaffected). If QPs were to explain the plateau in TLS *Q* factor, then this analysis predicts a QP saturation temperature of approximately 130 mK. This is consistent with recent studies (*47*–*49*) of nonequilibrium QP population in Al SC circuits, which infer a QP population with effective temperature of 120 to 150 mK. Details of the QP loss model and further discussion of possible sources of thermally activated TLS relaxation channels are given in the “Dependence of relaxation on temperature” and “Possible relaxation channels for TLS” sections in the Supplementary Materials and also in (*50*). We emphasize that this effective temperature captures the excess out-of-equilibrium QP density, which is relevant for transmon and TLS relaxation. This should not be confused with the temperature of QP that governs QP’s energy distribution, which has been shown to be in equilibrium with the mixing chamber plate temperature (*51*). Last, we remark that TLS-QP coupling is simply one possible explanation for the observed temperature-dependent TLS relaxation behavior. This behavior deviates from predictions of the standard tunneling model of TLS (*22*) and reveals previously unexplored TLS physics that requires further investigation.

## DISCUSSION

Despite the success of using TLS as a phenomenological model, in-depth knowledge of the nature and origin of TLS remains elusive in many specific situations (*3*, *52*). The long-lived coherent microwave-frequency TLS realized in this work through phonon engineering of the host material should be able to shed new light on previous TLS studies.

The extended *T*_1_ time of the acoustically shielded microwave-frequency TLS, along with dynamical decoupling sequences, presents an opportunity to use it as highly sensitive nanoscale sensors (*53*, *54*) capable of revealing the structure of its local environment (*55*, *56*). Of particular interest are low-frequency TLS that can be thermally activated. These RF-frequency TLS exhibit fluctuations due to interactions with thermal phonons and have been linked to parameter fluctuations of SC qubits over extended timescales (*8*, *15*, *16*, *40*) and 1/*f* noise in conductors (*57*). These RF-frequency TLS are atomic-scale, and sensing them is only possible through another sensitive atomic-scale sensor, which is made available with the long-lived microwave-frequency TLS. In each of these cases, a better understanding of the TLS could potentially lead to strategies for their elimination through materials selection, growth, and processing (*3*, *58*–*60*).

Phonon engineering methods aiming at RF frequencies could work for these RF-frequency TLS as well, in a way similar to our demonstration here. By suppressing thermal phonons, one can quiet the fluctuations of these RF-frequency TLS, which would in turn stabilize SC qubit parameters. Because RF-frequency TLS are believed to dominate the *T*_2_ coherence time of microwave-frequency TLS, their stabilization could also lead to significant enhancement of the *T*_2_ of microwave-frequency TLS. This coherence time of the long-lived TLS could be further extended through dynamical decoupling, making them favorable candidates as qubits or quantum memories (*38*, *61*), with coherent control provided by SC devices such as the transmon qubit in this work. This would make the hybrid transmon-TLS system a quantum register, much similar to those explored with spin qubits (*62*), with an avenue for scaling up.

When it comes to building a long-lived SC qubit that leverages the enhanced TLS lifetime, one could look to reduce the number of TLS in the bath to make the TLS bath spectra discrete. This means building an electric qubit with a smaller volume shunt capacitor or with no shunt capacitor at all as in the mergemon (*36*). In that case (with a discrete TLS spectra), the increase in the qubit lifetime due to coupling to TLS will be strongly influenced by the TLS lifetime, which can be engineered as in this work. This is similar to the proposed design as in (*63*), and an acoustic version has been experimentally demonstrated in (*13*). There is also the opportunity to further extend the TLS *T*_1_ lifetime using the acoustic engineering approach of this work. Estimates of the phonon-bath–limited TLS *T*_1_ time in an acoustic bandgap structure are in the seconds (*13*). As discussed above, the measured plateau in TLS energy relaxation time with temperature points to the possibility that QPs tunneling through the JJ may provide a secondary damping channel for TLS. This tunneling process, as shown recently, can be effectively suppressed by SC gap engineering (*64*).

Last, there is low-temperature flux noise found in SQUID loops, which limits the coherence of frequency-tunable SC qubits (*65*–*68*). This noise is hypothesized to arise from surface defects in the vicinity of the SQUID loop that carry spin degrees of freedom (*5*). This notion draws interesting connections between the TLS of amorphous solids and the color center defects of crystalline hosts such as diamond, which also experience a major source of noise from surface spins (*59*, *69*). Similarly, phonon engineering could mitigate unwanted phonon damping for acoustically active transitions of the defect center, such as found in the groundstate manifold of SiV (*70*, *71*).

## MATERIALS AND METHODS

### Device fabrication

Our fabrication process of the hybrid device stems from the fabrication recipe for transmon qubit on SOI substrate outlined in (*33*). Our modified process is illustrated in fig. S5. We start with an SOI wafer (SEH) with the following specifications: silicon device layer, 220 nm in thickness; resistivity, ρ ≥ 5 kΩ · cm; buried silicon dioxide layer, 3 μm in thickness; and a silicon handle, 750 μm in thickness, ρ ≥ 5 kΩ · cm. First, the wafer is diced along the 〈100〉 direction into chips of dimensions 20 mm by 10 mm. We then perform the following fabrication steps, all using 100-keV electron beam lithography (Raith EBPG5200) for patterning and electron beam evaporation (Plassys MEB 550S) for metalization: (i) Si device layer patterning using inductively coupled plasma reactive ion etching with C_4_F_8_/SF_6_ (Oxford Plasmalab 100) to define the cross-shield acoustic metamaterials and the release holes for device suspension; (ii) 30^∘^ double-angle evaporation for the Manhattan-style JJ (30/50 nm) using a single-layer photoresist (ZEP520A). The oxidation steps are performed at 130 mbar for a duration of 84′ and 102′ for Chip-A and Chip-B, respectively; (iii) Al ground plane patterning by liftoff; (iv) Ar ion milling, bandage deposition, and liftoff; and (v) device release in anhydrous vapor-HF (SPTS uEtch).

## Supplementary Materials

### This PDF file includes:

Supplementary Text Figs. S1 to S24 Tables S1 to S6 References
