# Supplementary material for: Phonon engineering of atomic-scale defects in superconducting quantum circuits
Source: Sci Adv. 2024 Sep 13;10(37):eado6240. doi: 10.1126/sciadv.ado6240 (PMC11397498; doi:10.1126/sciadv.ado6240)
Supplement: Supplementary file 1 — Supplementary Text Figs. S1 to S24 Tables S1 to S6 References [file sciadv.ado6240_sm.pdf]

Science Advances
AAAS

Supplementary Materials for

Phonon engineering of atomic-scale defects in superconducting quantum circuits

Mo Chen *et al.*

Corresponding author: Oskar Painter, opainter@caltech.edu

Sci. Adv. **10**, eado6240 (2024)
DOI: 10.1126/sciadv.ado6240

This PDF file includes:

Supplementary Text
Tables S1 to S6
Figs. S1 to S24
References

I. METHODS

In this section, we describe considerations that underlie the design of the hybrid transmon qubit device with Josephson junctions (JJs) embedded in acoustic bandgap structure. Our overarching goal is to strike a balance between the simplicity of the transmon qubit device and its effectiveness in demonstrating the phonon engineering of tunneling two-level systems (TLS) defects. This guiding principle is reflected in our decision of excluding Purcell filters, as well as the inclusion of a shunt capacitor for the transmon qubit. Further discussions on the device design will follow shortly. Along with the transmon qubit device design, we consider the acoustic metamaterials, as well as their integration into the transmon device. We will also discuss the device fabrication process, wherein a single resist layer Manhattan-style Josephson junction process plays a key role in the realization of our device. To conclude the Methods section, we provide brief descriptions of our experimental measurement setup, the calculation of phonon density of states using COMSOL, and a technique we used to generate new sets of TLS, known as thermal cycling.

A. Device design

The device serves two purposes: a) to identify individual TLS influenced by the engineered acoustic environment, and b) to characterize their relaxation behavior. To achieve this, we direct our attention towards TLS that are physically located inside the Josephson junction (JJ) tunnel barriers. This choice has three advantages. Firstly, their strong couplings to the transmon qubit, due to the intense electric field within the JJ, set them apart from TLS at circuit interfaces. Secondly, their physical confinement within a small area (the JJ) makes it convenient for phonon engineering. Lastly, individual addressing and characterization of TLS inside the JJ are well-established[37,38,41].

1. *Transmon qubit*

In the design of the transmon qubit, we decide to make our JJ an order-of-magnitude larger than typical JJs, with a size of approximately $\sim 0.83\ \mu\text{m}^2$, in order to increase occurrences of TLS inside the JJ. Such large JJ contributes to a substantial junction capacitance. A transmon qubit, characterized by its qubit capacitance consisting mainly of the junction capacitance, has been recently demonstrated in refs.[35,36]. In our design, the two JJs that form a symmetric SQUID (superconducting quantum interference device) loop collectively contribute a ~ 60 fF junction capacitance to the transmon qubit. It is noteworthy that the junction capacitance is large enough that the JJs alone can make up a transmon qubit, a configuration termed the 'merged-element transmon'[35,36]. In this work, however, instead of implementing a full merged-element transmon, we introduce a shunt capacitor. The shunt capacitor conveniently facilitates coupling to control lines and readout resonators. The shunt capacitor accounts for ~ 40 fF, resulting in a total transmon capacitance of ~ 100 fF. It is important to emphasize that the shunt capacitor is not protected by the acoustic metamaterials, and its interaction with nearby resonant TLS is considered the major T_1 relaxation channel for the transmon qubit. Consequently, due to the influence of the shunt capacitor, we do not expect substantial effects of phonon engineering on the transmon qubit in the current design.

To engineer the acoustic environment that the JJ and the TLS inside the JJ see, we position the JJ of the transmon qubit on top of a rectangular platfrom consisting of an unpatterened Si suspended membrane, as shown in Fig. S1. The rectangular platform is tethered to the rest of the Si microchip through an acoustic metamaterial, which is designed to exhibit a microwave-frequency acoustic bandgap centered around 5.1 GHz. Inside the bandgap, the acoustic metamaterial shields the TLS from spontaneous phonon emission into the phonon modes of the bulk materials and extends the lifetime of TLS.

The readout resonators are designed to situate ~ 700 MHz above the transmon's upper sweet spot frequency, with a coupling strength of ~ 70 MHz and a linewidth of ~ 2 MHz. No Purcell filters are used, yielding a Purcell limit of $\sim 10\ \mu$s, a timescale that is on the same order of transmon's T_1. We believe the Purcell limit serves as the secondary contribution to the relaxation process of transmon, with the major contribution being resonant coupling to TLS at the shunt capacitor, as mentioned earlier.

For the comprehensive characterization of phonon engineering of TLS and the acoustic bandgap, two distinct microchips are designed, labelled Chip-A and Chip-B. Each chip accommodates four transmon qubits. On Chip-A, the designed upper sweet spot frequencies of the four transmon qubits span the range of $6 - 6.5$ GHz, strategically chosen to resolve the upper edge of the acoustic bandgap. Conversely, the four transmon qubits on Chip-B are designed to cover the frequency range $5.1 - 5.5$ GHz to resolve the lower edge of the acoustic bandgap. On each chip, the four transmon qubits have identical JJs. Adjustments to the shunt capacitance of each transmon qubit tunes its sweet spot frequency to the desired value. Between Chip-A and Chip-B, the geometry remains identical. The

different transmon frequency ranges between the two chips are achieved by varying the oxidation condition during the JJ fabrication process.

The fabricated Chip-A (Chip-B) covers upper sweet spot frequency ranges 6–6.5 GHz (5.4–5.8 GHz). Typical parameters for all transmon qubits on both chips are presented in Table S1. It's worth noting that these parameters are subject to minor changes during different cool downs. On Chip-B, Q_4 remains operational, indicated by the Lamb shift of the readout resonator and its susceptibility to flux tuning via the crosstalk from the Z lines of Q_{1-3}. However, Q_4 does not show frequency tuning through its own Z line. We suspect that potential defects in the Z line or associated wirebonds lead to an open connection. We therefore exclude Q_4 from Chip-B in this study. Parameters of the other seven transmon qubits align well with our design and simulations. Their T_1 times span the range 1.5–6.0 μs, corresponding to Q values between 0.5–2 $\times 10^5$, which are on par with the best SOI transmon qubits reported in literature[33]. Fig. S2 displays a representative Rabi chevron pattern measured on Q_4 of Chip-A.

2. *Acoustic bandgap metamaterials*

The acoustic metamaterial is formed from a periodic cross-shield pattern etched into the Si membrane layer. A scanning electron microscope (SEM) image showcasing the JJ, the rectangular platform, and the surrounding acoustic metamaterials is shown in Fig. S1a. The platform region replaces the central 2×3 unit cells of the cross-shield metamaterial, and is surrounded by nine periods of shielding. As shown in the SEM of the device (Fig. S1a) and the schematic (Fig. S1b), the JJ leads pass through several unit cells of the acoustic metamaterial, introducing additional mass and perturbations to the band structures. Consequently, central to our design is the establishment of a large acoustic bandgap in the presence of the JJ leads.

To account for these perturbations due to the JJ leads, we consider three different unit cell types in the COMSOL simulation of the acoustic band structure. These unit cell types are designed in accordance with the geometry of our transmon device, which include **a** silicon only (enclosed by the blue dashed box in Fig. S1b), **b** silicon with 30 nm thick Al leads from the JJ (enclosed by the red dashed box in Fig. S1b), and **c** silicon with 50 nm thick Al leads from the JJ (enclosed by the green dashed box in Fig. S1b). Throughout all three cases, the Si geometry stays the same. The dimensions used by the COMSOL simulations are derived from the SEM image of a sister chip that is nominally identical to Chip-B. The results from the COMSOL simulations for the acoustic band structures in these three scenarios, along special paths connecting highly symmetric points in the k-space, are shown in Fig. S3. Additionally, the corresponding bandedge frequencies are listed in Table S2. The overlap of these three simulated bandgaps yields an overall bandgap spanning 1.372 GHz, ranging from 4.442 GHz to 5.814 GHz, in the presence of the JJ leads. The overall bandgap is 0.219 GHz narrower than the Si only unit cell (4.442–6.033 GHz), due to the perturbations of Al leads.

Additionally, we conducted COMSOL simulations to explore the effects of the widths and thicknesses of the Al leads that run through the Si cross-shield pattern. Of the two factors, thickness has the larger impact on the band structures. As the thickness of the Al lead increases, the size of the bandgap decreases from 1.591 GHz until it vanishes at approximately 100 nm Al thickness. Preserving a large acoustic bandgap therefore necessitates careful design of the JJ geometry and the process of double-angle evaporation of Al. It is critical to ensure that any cross-shield metamaterial unit cell undergoes no more than one metalization, or equivalently, avoiding the formation of parasitic junctions on the cross-shield pattern. This condition ensures that the three scenarios simulated in Fig. S3 faithfully capture all the acoustic band structures encountered in our device. The elimination of parasitic junctions in the cross-shield region is achieved by a geometric argument, which will be discussed in detail in the subsequent section on device fabrication (Sec. I B).

3. *Localized acoustic phonon modes in the bandgap*

In the center of the acoustic bandgap metamaterial, 2×3 unit cells are replaced by a suspended unpatterned Si rectangular platform, on top of which the JJ sits. The platform region has a size comparable to the acoustic wavelength, therefore supporting a few localized acoustic modes inside the acoustic bandgap frequency range. Through design, we minimized the platform area needed for the junction angled evaporation, as detailed in Sec. I B 3. This minimal Si platform and the junction structure on top of it minimizes the total number of localized acoustic modes.

Accurate simulation of these modes are demanding, due to the large total area involved and small feature size of the device geometry. We estimated the localized acoustic modes in COMSOL. The frequency and quality factor of simulated eigenmodes are shown in Fig. S4, considering a structure of the Si platform, surrounding Si acoustic bandgap metamaterial, and aluminum JJ on top. There is a set of 10 discrete localized acoustic modes, represented by open circles, with a free spectral range on the order of 100 MHz, and quality factors on the order of 10^7 in Fig. S4.

To ensure that we are not just measuring these long-lived harmonic acoustic modes, we have checked the anharmonicity of the detected TLS states. As discussed in more detail in Sec. II F, the TLS we measured are effectively two-level systems with very large anharmonicity, resolving the concern that these detected states are high-Q harmonic acoustic modes. However, these local acoustic modes could still play a role when they hybridize with TLS. In this case, the measured strong anharmonicity of TLS indicates that they would still be predominantly TLS-like hybrid modes.

B. Device fabrication

Our fabrication process of the hybrid device stems from the fabrication recipe for transmon qubit on silicon-on-insulator (SOI) substrate outlined in ref.[33]. Our modified process is illustrated in Fig. S5. We start with an SOI wafer (SEH) with the following specifications: silicon device layer, 220 nm in thickness, resistivity $\rho \geq 5$ k$\Omega \cdot$ cm; buried silicon dioxide layer, 3 μm in thickness; and a silicon handle, 750 μm in thickness, $\rho \geq 5$ k$\Omega \cdot$ cm. First, the wafer is diced along the $\langle 100 \rangle$ direction into chips of dimensions 20 mm $\times$ 10 mm. We then perform the following fabrication steps, all using 100 keV electron-beam lithography (Raith EBPG5200) for patterning, and electron beam evaporation (Plassys MEB 550S) for metalization: (i) Si device layer patterning using inductively coupled plasma reactive ion etching (ICP-RIE) with C_4F_8/SF_6 (Oxford Plasmalab 100) to define the cross-shield acoustic metamaterials, as well as the release holes for device suspension. (ii) 30° double-angle evaporation for the Manhattan-style JJ (30 nm/50 nm) using a single layer photo resist (ZEP520A). The oxidation steps are performed at 130 mbar, for a duration of $84'$ and $102'$ for Chip-A and Chip-B, respectively. (iii) Al ground plane patterning by liftoff. (iv) Ar ion milling, bandage deposition and liftoff. (v) Device release in anhydrous vapor-HF (SPTS uEtch).

Our fabrication process is fine-tuned to accurately realize our design and ensure the preservation of a large acoustic bandgap. The key in our fabrication is to minimize any perturbations to the acoustic structure, in particular: a) Preventing any metal deposition on the vertical sidewalls of the silicon acoustic structures, and b) eliminating the formation of parasitic junctions on the silicon cross-shield structures during the double-angle evaporation process.

1. *Preventing metal deposition on the Si sidewalls*

To avoid any undesired metal deposition on the vertical sidewalls of the silicon acoustic structures, we make sure 1. the JJ lead is small enough to completely locate on top of the Si structure, and 2. there is good alignment between the JJ and acoustic metamaterial patterns. For the first part, we make the width of the JJ leads narrower than the width of the Si tether, which is the narrowest part of the cross-shield acoustic metamaterial that the JJ lead runs through, given by $w_{\text{lead}} = 45$ nm $< w_{\text{tether}} = 72$ nm, as illustrated in Fig. S1b. For the second part, we employed local markers during the e-beam lithography, which contributes to consistent alignment accuracy, resulting in small misalignment of $\lesssim 10$ nm between the acoustic metamaterial pattern and the JJ, as evidenced in Fig. S1a. Even considering a worst case scenario with a 10 nm misalignment, the metal deposition of the JJ leads remains confined to the top silicon surface, avoiding any undesired metal deposition onto the sides of the silicon acoustic structures.

2. *Eliminating the formation of parasitic junctions*

To eliminate the formation of parasitic junctions on the cross-shield metamaterials, we implement a geometric strategy in the Manhattan-style JJ, as illutrated in Fig. S6. During the angled evaporation process, an unmetalized 'shadow' area of size $l_{\text{shadow}} = d \tan\theta$ is created, where d is the thickness of photoresist and θ the evaporation angle from normal incidence. In our process, the shadow size is approximately $l_{\text{shadow}} \approx 150$ nm. In the design of the Manhattan-style JJ, we enforce the condition that $w_{\text{lead}} \ll l_{\text{shadow}}$, where $w_{\text{lead}} = 45$ nm is the width of our JJ leads. This condition guarantees that only one layer of Al is metalized on the Si structure, avoiding the formation of parasitic junctions in the Manhattan-style JJ configuration. Specifically, the fabrication of Manhattan-style JJ involves two separate Al evaporations, whose in-plane evaporation angles are perpendicular to each other. When the in-plane direction of the evaporation aligns with the length direction of the JJ lead (into the plane in Fig. S6), Al is deposited to the Si structure. When the in-plane direction of the evaporation is perpendicular to the length direction of the JJ lead, as shown in Fig. S6b, Al only deposits onto the resist, and is subsequently lifted off. Consequently, no parasitic junctions are formed during the double-angle evaporation process. This is important to the preservation of a large acoustic bandgap, as excessive Al deposition on the cross-shield acoustic metamaterial can quickly diminish the bandgap, as discussed previously.

We note that parasitic junctions still exist in our fabrication process, where we broaden up the JJ leads for the bandage. However, these parasitic junctions are strategically positioned outside the acoustic metamaterial region. As a result, the increased thickness and weight of Al in these areas do not affect the acoustic bandgap. These parasitic junctions are shorted by a bandage at the final stage of the fabrication (step v). We remark that for a single JJ qubit (fixed frequency qubit), it is indeed possible to completely eliminate the parasitic junctions, through purely geometric considerations. This is important in future work when we embed the whole merged-element transmon qubit[35,36] into the acoustic structure and remove the shunt capacitor from our design.

3. *Single resist layer JJ process*

In conjunction with the criterion $w_{\mathrm{lead}} \ll l_{\mathrm{shadow}}$, we make an additional effort to minimize the size of the shadow area l_{shadow}. The shadow area is inherently part of the rectangular platform on which the JJ resides, as depicted in Fig. S1. As such, a larger shadow region requires a larger rectangular platform. A larger platform in turn supports more localized acoustic phonon modes inside the acoustic bandgap, which might potentially influence TLS performance.

In order to suppress the number of these localized acoustic phonon modes, and avoid their potential couplings to TLS, we have developed a single resist layer JJ fabrication process, similar to that outlined in ref.[72], using ZEP520A instead of the more conventional PMMA-MMA double layer resist process. This process minimizes the size of the shadow area to $l_{\mathrm{shadow}} \approx 150$ nm, resulting in free spectral range of the localized acoustic phonon modes within the acoustic bandgap on the order of ~ 100 MHz according to COMSOL simulations shown in Sec. I A 2.

It is important to note, however, that we have noticed the formation of free-standing vertical Al sidewalls post the liftoff process in some of our devices, as shown in Fig. S7. This phenomenon is anticipated when single layer resist is used in angled evaporation, without an undercut. In this scenario, the metal deposited on the sidewall of the resist, as indicated in Fig. S6a, might not be entirely removed through the liftoff process. However, despite the presence of these residual vertical Al sidewalls, we have not observed impacts on the performance of the transmon qubits.

4. *JJ oxidation*

The oxidation condition for the merged-element-style JJs are determined based on measurements of previous calibration chips, as shown in Fig. S8. An extended oxidation duration at high static oxygen pressure grows a thicker AlO_x barrier layer of the JJ, which decreases the Josephson energy E_J and the transmon frequency. Calibration data show an empirical linear dependence between the transmon frequency and the oxidation duration, to which we fit and inform our fabrication of Chip-A and Chip-B. The one outlier is a chip that aged for approximately one month prior to measurement, which explains the atypical behavior. The frequencies of fabricated Q_1's of Chip-A and Chip-B, represented by pentagrams agree well with the empirical linear fit.

C. Measurement setup

Fig. S9 shows a schematic of the measurement setup inside the cryogen-free dilution refrigerator (Bluefors LD400), which includes standard shielding and filtering for superconducting transmon qubit experiments[73,74]. The refrigerator consists of multiple temperature stages, which in descending order of temperature are 300 K, 50 K, 4 K, still, cold plate (CP), and mixing chamber (MXC) flanges. The experimental sample is mounted to the MXC plate. Under standard operating conditions, the MXC plate achieves a base temperature of 7 mK, providing the low temperature environment required for the experiments.

The frequency control of each transmon qubit is achieved by a bias current that generates a magnetic field threading through the SQUID loop of the transmon qubit. The bias current consists of two parts: the static DC bias (slow Z) and the dynamic RF pulse (fast Z). The static DC bias is generated by a stable DC voltage source (QDevil QDAC) passing through a 2.8 kΩ resistor at room temperature. The DC current is filtered by a RC low-pass filter (QDevil QFilter) at 65 kHz placed at the 4 K stage. The DC bias provides a broad tuning range and high tuning precision for the transmon qubit frequency. The static DC bias is combined with a dynamic RF pulse (fast Z) through a DC-coupled bias tee (Mini-Circuits ZFBT-4R2GW+ with the capacitor shorted). The fast Z pulse is generated directly by an arbitrary waveform generator (AWG, Keysight M3202A), introducing dynamic tuning capabilities for the transmon qubit frequency. For the present experiment, we have not performed corrections for Z line distortions, as discussed in ref.[75]. Consequently, a slight drift in the patterns of vacuum Rabi oscillations at short Z duration is observed, as seen in Fig. 2 of the main text and Fig. S19.

The resonant control of transmon qubit is achieved by the XY line, which couples capacitively to the transmon qubit through a coupling capacitance of approximately ~ 80 aF. We use a total of 50 dB attenuation (XMA cryogenic attenuators) in the fridge XY lines, to accommodate the need of higher microwave driving power for the direct control of TLS (to be discussed in Sec. III 1). The microwave signal is generated at room temperature. A pair of intermediate frequency (IF) signals from the AWG (Keysight M3202A), in conjugation with a local oscillator (LO) signal from a microwave signal generator (Rhode&Schwarz SMB100A), undergoes IQ mixing (Marki Microwave MMIQ-0218L) and generates a single sideband microwave signal that achieves the XY control of the transmon qubit as well as TLS.

For the readout (RO) of the transmon qubits, a microwave RO input signal (generated and filtered similarly to the XY signal) is passed down to the feedline of the sample. The RO output signal from the feedline is first amplified by a JTWPA (Josephson traveling wave parametric amplifier) which is sandwiched by two sets of circulators (Low Noise Factory LNF-CICI4_12), a HEMT (high electron mobility transistor, Low Noise Factory LNF-LNC4_16B or LNF-LNC0.3_14A) amplifer, a low-noise room temperature amplifier (MITEQ LNA-30-0400800-07-10P), a high pass-filter (Mini-Circuits VHF-4600+), a tunable attenuator (Vaunix Lab Brick LDA-133), and another MITEQ low-noise amplifier (MITEQ LNA-30-0400800-07-10P). The RO output signal is then downconverted at room temperature by an IQ mixer and the same LO used to generate the RO input signal. The resulting in-phase (I) and quadrature (Q) signals are filtered (Mini-Circuits VLF-160+), amplified (Mini-Circuits ZFL-500HLNB+), and digitized (Keysight M3102A) for qubit readout. In addition to the aforementioned filtering, low-pass filters (Mini-Circuits VLFX-400+, K&L Microwave 6L250-12000/T26000) and infrared Eccosorb filters (custom made) are added at the MXC plate where appropriate. All the microwave instruments are synchronized to an external 10 MHz reference clock from a Rubidium frequency standard (Stanford Research Systems FS725). The AWG and digitizer are both triggered by a delay generator (Stanford Research Systems DG645).

D. Phonon density of states

In this section we describe the process of finding the phonon density of states (DOS) based on the COMSOL simulated band structures. To achieve this, we expand upon the simulations presented in Fig. S3, which focus on special paths connecting points of high degrees of symmetry. These simulations are efficient in finding the bandgap frequencies. However, they do not represent the entire band structures, and consequently, the phonon DOS. To extract the phonon DOS, we leverage symmetries in our structure and uniformly sample one quarter of the first Brilloin zone in the two-dimensional k-space, given by $k_x, k_y \in [0, \pi/a]$, using N steps for the k_x, k_y values. Here, a denotes the length of the square unit cell. We then count the total number of k states in the first Brillion zone, accounting for symmetries. The results are then grouped into frequency bins of 80 MHz interval based on the frequencies of the eigenstates, and normalized by a factor of $1/(2N-2)^2$ to arrive at the phonon DOS. The resulting phonon DOS for all three unit cell types is shown in Fig. S10, and a zoom-in view is displayed in Fig. 3e in the main text.

E. Thermal cycling of the device

In our experiment, we employ a method known as thermal cycling to generate new distributions of TLS on the same devices. This method has been shown to be effective in ref.[39] when the MXC plate temperature rises above ~ 20 K. In this study, we perform thermal cycling of the fridge to room temperature, then back down to the base temperature, to ensure the absence of correlation between the two sets of TLS characterized during different cool-down cycles.

II. DISCUSSIONS

In this section we provide analysis and discussions regarding the phonon engineering of TLS. We start by presenting a comprehensive list of parameters for all 56 TLS characterized and analyzing their distributions. Next, we derive the frequencies of the average acoustic bandgap, shared across all devices, driven by the TLS data. This is followed by an examination of individual devices, where we identify the distinctive acoustic bandgaps corresponding to each of the seven fabricated devices. The results extend and complement the data presented in the main text. Additionally, this analysis unveils disorders in the frequencies of individual device bandgaps, providing an explanation for some of the outlier data points mentioned in the main text. We then provide an explanation for the significant variations observed in the TLS T_1 relaxation times, based on the confined geometry of the device, thereby addressing the remaining outlier data points mentioned in the main text. Following this, we present experiments and data that corroborates the anharmonicity of TLS. Our experimental findings suggest that TLS is highly anharmonic. Intriguingly, our data also implies a three-mode coupling involving TLS, the transmon qubit, and an additional TLS. Furthermore, we present

data and analysis on the temperature-dependent relaxation of both the transmon qubit and TLS. This motivates a detailed discussion on possible relaxation channels for the TLS, offering a comprehensive view on the temperature-dependent TLS relaxation. Finally, we showcase direct XY control for TLS, which has been used to characterize both the energy relaxation T_1 and dephasing T_2^* of TLS. The result raises intriguing questions regarding the interactions between quasiparticles and TLS.

A. TLS parameters

A complete list of TLS frequencies, their respective coupling strengths g to the transmon qubit, and T_1 relaxation times, measured on Chip-A and Chip-B, is provided in Table S3 and Table S4, respectively. This dataset that includes 56 distinct TLS has been acquired across seven transmon devices. The TLS characterized span frequencies from 3.7421 GHz to 6.3935 GHz, and their T_1 values range from 0.25 ± 0.02 μs to 5400 ± 800 μs (Fig. S11). The TLS frequencies and coupling strengths g are extracted through fitting the avoided crossings in the microwave spectroscopy of transmon qubits to the transmon-TLS interaction model

$$\begin{aligned} \mathcal{H} &= \frac{\omega_q}{2}\hat{\sigma}_q^z + \frac{\omega_{\mathrm{TLS}}}{2}\hat{\sigma}_{\mathrm{TLS}}^z + \hat{H}_{\mathrm{int}}, \\ \mathcal{H}_{\mathrm{int}} &= g(\hat{\sigma}_q^+\hat{\sigma}_{\mathrm{TLS}}^- + \hat{\sigma}_q^-\hat{\sigma}_{\mathrm{TLS}}^+), \end{aligned} \tag{S1}$$

where ω denote their frequencies, $\hat{\sigma}^z, \hat{\sigma}^\pm$ are the Pauli operators.

We remark that certain TLS T_1's measurements are conducted using a strong microwave pulse that directly drives the TLS to its excited-state. These particular TLS T_1 values are distinctly marked by † in both Table S3 and Table S4). We note that the T_1 values obtained by this method appear comparatively shorter than those measured using SWAP with the transmon qubit. Moreover, TLS relaxation curves measured by this method can sometimes deviate from a simple exponential decay. When such deviations are evident, we report the relaxation values derived from fitting to a double exponential model (Eq. S36). We attribute both phenomenon to interactions with the quasiparticles (QP) induced by the strong microwave pulse[76,77], which will be discussed later in Sec. III 3.

During the TLS T_1 measurements, the transmon qubit also acts as an energy dissipation channel for the TLS, inducing a Purcell decay on the TLS T_1 lifetime. The Purcell-limit follows $(g^2/\Delta^2) \cdot \Gamma_{1,q}$, where g is the TLS-transmon coupling strength, Δ their frequency detuning during the T_1 relaxation, and $\Gamma_{1,q} = 1/T_{1,q}$ the relaxation rate of the transmon qubit. When measuring TLS T_1 relaxation, the transmon qubit is usually tuned to its upper sweet spot, away from the TLS, to maximize the Purcell-limit. There are a few cases when the transmon at the upper sweet spot might still impose a Purcell-limit. One such case is for TLS18–23 (Chip A Q_3 during CD1), where qubit upper sweet spot (6.11 GHz) hybridizes with TLS18 (6.0877 GHz) with a coupling strength $g = 30.2$ MHz. To increase the Purcell-limit, we tuned the transmon to 5.799 GHz during TLS T_1 measurements. We then used a fast flux to tune the transmon back to the upper sweet spot for readout. Another case is for TLS35 (Chip B Q_1 during CD1), where we DC flux tuned the transmon qubit to 5.454 GHz.

In Fig. S12 we show the ratio between the estimated Purcell-limit and the measured T_1 lifetime for each TLS, represented by blue crosses. TLS35 exhibits $T_1 = 261 \pm 21$ μs, close to the Purcell limit from the transmon qubit (Q_1 of Chip-B). In light of this proximity to the Purcell limit, and the likelihood that the measured TLS35 T_1 value falls short of its intrinsic T_1, TLS35 is excluded from all the median and mean T_1 statistics. Among the remaining 55 TLS characterized, their Purcell limits from the transmon are considerably higher than their measured relaxation times, by at least a factor of 3×, indicated by the red dashed line as a guide for the eye. As such, the T_1's of these 55 TLS are likely not Purcell-limited, and they are all used in the calculation of the median and mean values reported in this study.

B. TLS distributions

In this section, we analyze three distributions of the TLS parameters: 1. the distribution of TLS T_1, 2. the distribution of their coupling strengths g to the transmon qubit, and 3. the distribution of TLS T_1 against g.

1. *TLS T_1 distribution*

To begin, we look at the distribution of TLS T_1 from all 55 TLS, in supplementary to Fig. 3c in the main text. In Fig. S13, we present the cumulative distribution of TLS T_1 values for family A (blue squares) and family B

(red triangles), respectively. To characterize this distribution, we employ three commonly used models: the normal distribution, the exponential distribution, and the log-normal distribution. These models are given by their cumulative distribution functions (CDF),

$$\begin{aligned} \mathrm{CDF_{norm}}(x) &= \frac{1}{2}[1+\mathrm{erf}(\frac{x-\mu}{\sqrt{2}\sigma})], \\ \mathrm{CDF_{exp}}(x) &= 1-\exp(-\lambda x), \\ \mathrm{CDF_{logn}}(x) &= \frac{1}{2}[1+\mathrm{erf}(\frac{\ln x-\mu}{\sqrt{2}\sigma})]. \end{aligned} \tag{S2}$$

Based on the fittings using the three models in Fig. S13, represented by the solid lines, we identify the log-normal distribution as the best representation for our data. The resulting parameters yield distinct median T_1 values of 4.1 ± 0.2 μs for family A and 414 ± 17 μs for family B. These fitted values are consistent with those outlined in Table S5 based on the frequencies of the acoustic bandgap, which will be discussed later.

2. *TLS coupling strength distribution*

Next, we show the distribution of TLS coupling strengths g to the transmon qubit in Fig. S14. According to the standard tunneling model (STM), this distribution is a reflection of the electric dipoles of TLS, which has a density of[37]

$$d^2N/dEdg = \sigma A\sqrt{1-g^2/g_{\mathrm{max}}^2}/g, \tag{S3}$$

where E, A, σ are the energy of TLS, the area of the JJ, and the TLS density, respectively. The measured TLS distribution over coupling strength g overall aligns well with STM predictions. To determine the TLS density σ, we normalize the fitted parameter by the total size of JJ in the transmon qubit, which is approximately 1.66 μm^2, and the collective frequency span of the seven transmons in our search for these TLS, which amounts to 22 GHz. This results in a TLS density of $\sigma = 0.6$ GHz$^{-1}\mu$m^{-2}, in agreement with literature[24,36,37].

3. *TLS T_1 distribution over coupling strength*

In addition, the STM ascribes TLS relaxation to spontaneous phonon emission[1,2] via the interaction between TLS' elastic dipole and the acoustic environment. In this context, the TLS' elastic dipole is proportional to its electric dipole, governed by $\propto \Delta_0/E \propto \vec{d}$. Here Δ_0 is the tunneling energy, E the eigenenergy, and $\vec{d}$ the electric dipole of the TLS. As discussed above, the coupling strength g reflects the electric dipole of TLS. Consequently, a power-law dependence of $1/T_1 \propto g^\alpha$ is expected, and has indeed been observed for TLS located inside the Josephson Junctions of a phase qubit[39].

We remark that this power-law dependence is not unique to the spontaneous phonon emission process. Based on the STM, any relaxation process mediated through either the electric or elastic dipole of TLS would yield a power-law dependence. Therefore, it could also apply to our device, where the spontaneous phonon emission has been suppressed. In Fig. S15 we show the TLS T_1 distribution against their coupling strengths g to the transmon qubit. Here, the blue and red filled circles respectively represent TLS located outside and inside the average acoustic bandgap. At first glance, our data does not readily exhibit a clear power-law dependence for TLS either within or outside the average acoustic bandgap, partly due to the wide spread of the T_1 data points that obscures any underlying correlations.

To address this, we follow the method in ref.[39], and group the data into bins based on their g values. We then compute the mean and standard deviation in each bin, which are represented by the open markers and their errorbars, respectively, with corresponding colors in Fig. S15. These data points reveal a trend of negative correlation between the mean T_1 values and the coupling strength g, in alignment with expectations from the STM. However, this trend does not convincingly conform to a power-law dependence. We attribute this deviation to the relatively limited size of our available dataset. Additionally, the deviation could arise from the extreme ways in which the acoustic bandgap metamaterial structures the acoustic environment. This influence can even extend to frequencies outside the acoustic bandgap. In such cases, the substantial alteration in the acoustic DOS, rather than the susceptibility to the acoustic environment, may prevail in determining the TLS T_1 distribution.

C. Identification of the average acoustic bandgap

As described in the main text, we select a T_1 cutoff between 35 μs and 85 μs to categorize all TLS into two groups: family A, characterized by shorter TLS T_1, and family B, characterized by longer TLS T_1. Remarkably, we observe a strong correlation between this categorization based solely on T_1 values and the frequency distribution of TLS within the two families. This correlation motivates us to identify an average acoustic bandgap across all seven transmon devices, using the following cost function,

$$\mathcal{C}(f_1, f_2) = \log[1 - F_A(f_1, f_2) \times F_B(f_1, f_2)], \tag{S4}$$

where the frequency band is specified between f_1 and f_2. $F_A(f_1, f_2)$ denotes the fraction of TLS in family A whose frequencies lie outside this defined frequency band, while $F_B(f_1, f_2)$ represents the fraction of TLS in family B that fall within this frequency band.

The landscape of the cost function $\mathcal{C}(f_1, f_2)$ is provided in Fig. S16 as a function of the lower bandedge frequency f_1 and upper bandedge frequency f_2. The minimum in the landscape yields $\mathcal{C}_{\text{min}} = -1.98$, which identifies the average acoustic bandgap present across all seven transmon devices. This average bandgap is characterized by

$$\begin{aligned} f_{1,\text{avg.bg}} &\in [4.510, 4.547] \text{ GHz}, \\ f_{2,\text{avg.bg}} &\in [5.690, 5.735] \text{ GHz}. \end{aligned} \tag{S5}$$

This average bandgap, in turn, yields a median TLS T_1 of $M_{\text{out,2D}}(T_1) = 4.4$ μs outside the bandgap and $M_{\text{in,2D}}(T_1) =$ 505 μs inside the bandgap. Our preference for using median over mean is justified by the large skewness for the TLS T_1 distributions.

It's important to note that the average bandgap, shared across different fabricated devices and chips, represents a lower-bound estimate, due to fabrication disorder on individual devices, which will be discussed shortly in Sec. II D. Despite this, the average acoustic bandgap still boasts a width exceeding 1 GHz. Furthermore, it exhibits a remarkable similarity to the COMSOL simulated bandgap, differing by merely $\lesssim$ 100 MHz, as shown in Table S5. This high degree of agreement underscores the reproducibility and robustness of the overall fabrication process for the acoustic bandgap metamaterial.

Lastly, we emphasize that the determination of the frequencies of the average acoustic bandgap does not depend on any a priori knowledge of the existence of an acoustic forbidden band. Instead, these bandgap frequencies arise naturally from the TLS data itself.

D. Acoustically-shielded TLS on individual devices

Using the full set of TLS data collected from all seven fabricated transmon devices, we present additional details complementing the information in Fig. 3 from the main text, and demonstrate the robust TLS T_1 enhancement on all devices from the acoustic bandgap. In Fig. S17 and Fig. S18, we present the TLS T_1 relaxation data measured on individual transmon devices, for Chip-A and Chip-B, respectively. These plots reveal the existence of two families of TLS, based on their frequencies and T_1 times, for each device. We use the same method for identifying the average acoustic bandgap to analyze the bandgaps of these individual devices. For each transmon device, we search for the frequency range of the acoustic bandgap $[f_1, f_2]$ that minimizes the cost function $\mathcal{C}(f_1, f_2)$. This analysis yields the frequencies of either one or both of the bandedges, depending on the frequency ranges and total number of TLS characterized on the particular transmon device. The determined frequencies of the bandedges f_1 and f_2 are depicted using gray shading in Fig. S17 and Fig. S18. For reference, we also plot the average bandgap frequencies $f_{1,\text{avg.bg}}$ and $f_{2,\text{avg.bg}}$ determined above in Sec. II C, using pink shading. The overlap between the individual device bandgaps and the average bandgap highlights the robustness of the fabrication process of acoustic bandgap metamaterial. These experimentally identified frequencies of the bandedges are listed in Table S5, along with the frequency range of the bandgap given by COMSOL simulations. The table also includes the median and mean TLS T_1 values both inside and outside the corresponding bandgaps.

Upon comparing the experimentally identified bandgaps across all seven devices, we observe disorder in the bandgap frequencies, which is most pronounced in Chip-A Q_2, as illustrated in Fig. S17b. In this case, we identify a bandgap that is up-shifted in frequency, which likely stems from fabrication disorder in the acoustic metamaterials. We remark that the upward shift in the bandgap frequencies for Chip-A Q_2 results in the TLS in family A, circled out in black in Fig. S17b, appearing as an outlier when using the average acoustic bandgap for analysis. However, when we apply the acoustic bandgap specific to this individual device, the TLS falls outside the bandgap, aligning with our expectation for family B. Similarly, we claim that in Fig. S17a, the TLS circled out on the left side is also misclassified as an

outlier when using the average acoustic bandgap. When using the acoustic bandgap of Chip-A Q_1, the frequency of this TLS (in family B) actually lies within the acoustic bandgap. We remark, however, that these analyses are based on limited data from individual devices, and we do not rule out other possibilities.

Regarding the remaining two outliers, marked by black circles in Fig. S17a and Fig. S18b, their frequencies locating outside the acoustic bandgap cannot be accounted for by the shift in individual device bandgap frequencies. It's worth noting that both of these outlier TLS belong to family B and exhibit long T_1 values, but reside outside the acoustic bandgap. We believe these TLS have either acoustic dipole orientations that are orthogonal to the polarization of the acoustic modes of the cross-shield structure, or, are decoupled from the acoustic bulk phonon modes of the SOI substrate due to the confined geometry of our device, which will be explained in Sec. II E. We note that a recent study [40] also reports such an outlier long-lived TLS in a device with no phonon engineering.

E. Variations in the relaxation time of TLS

The best fit to the log-normal distribution in Sec. II B suggests significant variations in TLS T_1 exceeding an order of magnitude. This phenomenon has been predicted and experimentally observed in various systems with confined geometries[22], including opto-mechanical cavities (OMC)[13] and nanomechanical resonators[10]. For example, numerical modeling in ref.[13] uncovered significant variations in the relaxation rate of both acoustic modes and TLS defects, spanning approximately two orders of magnitude both inside and outside the acoustic bandgap. We argue that this common characteristic in systems with confined geometries is also responsible for the large T_1 variations observed in our devices.

When a system is confined to a small scale, its thermal bath responsible for system relaxation is often composed of mesoscopic or even microscopic modes. As a result, these modes possess discrete frequencies in the frequency domain. Depending on the frequency of the system with respect to these discrete frequencies, the relaxation to the thermal bath modes can be dominantly resonant, or off-resonant, which significantly changes the relaxation rate. This interplay between the system's frequency and the discrete nature of the thermal bath modes contributes to the large variations observed in the system's (in this case, TLS) lifetime in confined geometries.

Let's delve into more details, and first consider the scenario where the TLS frequency lies outside the acoustic bandgap. In this case, the geometric limitations of the device results in discrete phonon modes across the frequency spectrum, with the free spectral range characteristic of the effective size of the system. As a consequence, TLS decays through both resonant and relaxation processes into neighboring discrete acoustic modes. The overall relaxation rate therefore heavily depends on the precise frequency configuration of both the TLS and the acoustic modes as well as their interaction strength. If the TLS frequency closely aligns with the resonance frequency of an acoustic mode, and the coupling between them is strong, the TLS will exhibit a fast relaxation rate. On the other hand, when the TLS frequency falls between neighboring acoustic modes, and their couplings are weak, the TLS will have slow relaxation rate. This results in large variations in the T_1 relaxation times of TLS outside the bandgap, which also indicates the presence of long-lived TLS outside the bandgap. These long-lived TLS outside the acoustic bandgap are predicted numerically in ref.[13], and have been observed experimentally in TLS7 and TLS51 of our device, which account for the outermost two outliers in Fig. 3d in the main text and in Fig. S17a, Fig. S18b.

The same argument applies to scenarios within the acoustic bandgap. Here, the TLS and the bulk/local phonon modes of the device exhibit larger frequency detunings on average. Consequently, TLS experience an even weaker relaxation from the interactions with acoustic modes. On average, this leads to prolonged TLS lifetimes inside the bandgap, but still with large variations in their T_1's.

In summary, the observed variations in TLS relaxation rate, as predicted by numerical modeling in ref.[13] and subsequently verified experimentally in this study, stem from the discrete nature of the thermal bath modes that govern the relaxation process. This discreteness in the thermal bath modes represents a common characteristic intrinsic to devices with a confined geometry.

F. Anharmonicity of TLS

1. *Two-excitation SWAP spectroscopy*

Let us now address a long-standing debate regarding whether coherent TLS are harmonic oscillator modes[3,78–80]. This question is of particular relevance in this study, because there potentially exist high-Q localized acoustic phonon modes, which may mimic TLS-like behaviors observed in our experiments (see also Sec. I A 3). We resolve this concern by demonstrating that individual TLS observed in our experiments become saturated with a single quanta of excitation, revealing their anharmonic nature.

The experimental sequence is illustrated in Fig. S19a. We initialize the TLS of interest in its excited-state, $|1\rangle$, and subsequently attempt to transfer a second quanta of excitation from the transmon qubit to this same TLS through SWAP spectroscopy. If TLS represents harmonic modes, it interacts with the transmon qubit via the interaction Hamiltonian

$$\mathcal{H}_{int} = g(\sigma^+ a + \sigma^- a^\dagger), \tag{S6}$$

where $\sigma^{+(-)}$ is the raising (lowering) operator for the transmon qubit, and a ($a^\dagger$) the annihilation (creation) operator for the harmonic mode associated with the TLS. In this context, the TLS harmonic mode would be able to absorb additional excitations at the same frequency (i.e. same flux bias of the transmon qubit in the SWAP spectroscopy) and climb up the Fock state ladder, according to the interaction Hamiltonian in Eq. S34. However, our experimental results, as shown in Fig. S19c–f for TLS1, TLS3, TLS4, and TLS5, reveal the absence of vacuum Rabi oscillations between the transmon qubit and excited-state TLS at the TLS frequency. The absence of vacuum Rabi oscillations unambiguously demonstrates that each TLS is fully saturated by a single quanta of excitation. This result establishes the anharmonic nature of all four characterized TLS. Furthermore, it validates that the TLS-like behavior we have characterized does not emerge from high-Q localized acoustic phonon modes supported by the rectangular platform region of our device.

We note that, to enhance the clarity of the TLS4 patterns, particularly in light of the overlapping TLS3 patterns, an additional step is taken in the experiment concerning TLS4 (Fig. S19e). In this experiment, we have prepared both TLS3 and TLS4 in their excited-states through sequential SWAP operations with the excited-state transmon qubit. Faint fringes from TLS3 are still visible in the obtained results, due to the T_1 relaxation of TLS3 back to its ground state while the preparation of TLS4 in the excited-state is in progress.

Furthermore, we conducted an extensive search across a wide frequency range for the potential $|1\rangle \leftrightarrow |2\rangle$ transitions of these four TLS. We compare the resulting SWAP spectroscopy of two excitations (Fig. S19c–f) to that of a single excitation (Fig. S19b). If the transition dipole moments of the $|1\rangle \leftrightarrow |2\rangle$ transition and the $|0\rangle \leftrightarrow |1\rangle$ transition are comparable, and the second excited-state of the TLS possesses a decent coherence time ($\gtrsim$ few hundred nanoseconds), we would anticipate the emergence of additional vacuum Rabi oscillations between the transmon and the second excitation of the TLS when they are on resonance. In particular, TLS3 and TLS5 exhibit a stronger coupling to the transmon qubit, $g \sim 20$ MHz, than the other two TLS. Given the larger value of g, it becomes easier to discern the presence of vacuum Rabi oscillations, particularly for low-frequency transitions, as the frequency resolution of the transmon, when functioning as a spectrometer, is limited by the amplitude resolution of the Z flux bias. This resolution tends to degrade as the transmon is tuned towards lower frequencies and becomes more flux sensitive.

In light of this, we selected TLS3 and TLS5 to conduct a broader frequency scan, extending up to 1.5 V of the flux bias. Notably, our scans up to a flux bias of 1.5 V did not reveal any additional vacuum Rabi oscillations. If TLS were to have a third state, these measurements would place bounds to the anharmonicity of the TLS, as listed in Table S6, where bound1 and bound2 denote the bounds for positive and negative anharmonicity, respectively, that satisfy $\alpha >$ bound1 > 0 or $\alpha <$ bound2 < 0. The data from all four TLS collectively indicate a conservative bound for the anharmonicity α, given by $\alpha > 0.41$ GHz or $\alpha < -1.3$ GHz, for positive and negative anharmonicity, respectively. Furthermore, the absence of emergent vacuum Rabi oscillations in the extensive scan range (0–1.5 V, corresponding to transmon frequencies of approximately 6.48–1.47 GHz) for TLS3 and TLS5 suggests that either TLS is highly anharmonic ($\alpha > 0.59$ GHz or $\alpha < -4.1$ GHz), or TLS has only two levels. It's important to note that all the data presented here were acquired on Chip-A Q_1 during CD2. While these TLS are designated TLS9–13 in Table S3, in this context, we refer to them as TLS1–5 to maintain consistency with the main text.

We would like to conclude this section by addressing a noticeable difference between the SWAP spectroscopy presented here (Fig. S19) and in Fig. 2d of the main text. This slight difference is attributed to the frequency shifts of TLS during the 200 mK thermal cycling in the temperature-dependent relaxation measurements, to be discussed in Sec. II G. The SWAP spectroscopy of two excitations was performed approximately two months after the initial single-excitation SWAP spectroscopy on the same qubit (Fig. 2 in the main text). In the meantime, thermal cycling up to 200 mK (Sec. II G) was carried out and lasted for over one month, inducing frequency shifts in all the TLS. We compare the TLS frequencies pre- and post-thermal cycling using the SWAP spectroscopy data (microwave spectroscopy was not taken post-thermal cycling). The comparison reveals that TLS1 frequency shifted by approximately $\gtrsim -100$ MHz, TLS3 by $\gtrsim -15$ MHz, TLS4 by $\gtrsim 40$ MHz, and TLS5 by $\gtrsim -10$ MHz. We note that TLS2 frequency drifted beyond the range of our scan, rendering us capable of only measuring the two-excitation SWAP spectroscopy for TLS1, TLS3, TLS4, and TLS5.

For comparison, the data in Fig. S19 were taken over the span of one month, and we did not observe noticeable frequency drifts of TLS1, 3, 4, 5 through SWAP spectroscopy. This suggests that the large frequency shifts were more likely provoked by elevated temperatures during the 200 mK thermal cycling, rather than being a sole consequence of the long time gap between measurements. Furthermore, when we tracked individual TLS frequencies for up to 90 hours at the 7 mK base temperature of the fridge (data not shown), we measured TLS frequency drifts in the range

of a few MHz, with the largest TLS frequency jump $\lesssim 2$ MHz. The observed TLS frequency jumps are significantly smaller than the frequency shifts of TLS1, 3, 4, 5, as described above. Notably, these few MHz frequency drifts are more than 10× smaller than reported in ref.[8], which is worth further investigation.

2. *TLS-transmon-TLS three-mode coupling*

In the SWAP spectroscopy of both Fig. 2 of the main text and Fig. S19, we have observed deviations from ideal chevron patterns, most noticeable for TLS3 and TLS5. These deviations are attributed to TLS-transmon-TLS three-mode couplings. This becomes clear when examining the Fourier transform of the single-excitation SWAP spectroscopy data, which reveals the frequencies of the vacuum Rabi oscillations, as shown in Fig. S20. Within the single-excitation manifold, the Hamiltonian governing the TLS-transmon coupled system can be expressed as follows:

$$\mathcal{H}_{\mathrm{TLS}-q} = \begin{pmatrix} \Delta/2 & g \\ g & -\Delta/2 \end{pmatrix}, \tag{S7}$$

where $\Delta = \omega_{\mathrm{TLS}} - \omega_q$ is the detuning between the transmon and the TLS, and g their coupling strength. Diagonalizing the Hamiltonian yields two eigenstates, featuring a frequency gap of $\sqrt{\Delta^2 + 4g^2}$. This particular frequency corresponds to the vacuum Rabi oscillations between the transmon and the TLS, which is captured by our single-excitation SWAP spectroscopy, shown in Fig. S20.

Now, we introduce the interaction of the transmon qubit with a second TLS. We expand the single-excitation manifold Hamiltonian in Eq. S7 to account for TLS-transmon-TLS three-mode coupling as follows

$$\mathcal{H}_{\mathrm{TLS}-q-\mathrm{TLS}} = \begin{pmatrix} \Delta_1 & g_1 & 0 \\ g_1 & 0 & g_2 \\ 0 & g_2 & \Delta_2 \end{pmatrix}, \tag{S8}$$

where Δ_1 (Δ_2) and g_1 (g_2) are the detuning and coupling strength between the first (second) TLS and the transmon qubit, respectively. Upon hybridizing the transmon qubit and the second TLS, the lower-right section of the Hamiltonian is block diagonalized, yielding

$$\mathcal{H}'_{\mathrm{TLS}-q-\mathrm{TLS}} = \begin{pmatrix} \Delta_1 & g_1 & 0 \\ g_1 & \omega_{h2}^- & 0 \\ 0 & 0 & \omega_{h2}^+ \end{pmatrix}, \tag{S9}$$

where $\omega_{h2}^{\pm} = \frac{1}{2}(\Delta_2 \pm \sqrt{\Delta_2^2 + 4g_2^2})$ are the eigenfrequencies of the hybridized transmon-TLS2 states. It's worth noting that the states associated with eigenfrequencies ω_{h2}^- and ω_{h2}^+ correspond to the bright and dark states, respectively. Further diagonalization of the Hamiltonian yields,

$$\mathcal{H}''_{\mathrm{TLS}-q-\mathrm{TLS}} = \begin{pmatrix} \omega_{h1}^+ & 0 & 0 \\ 0 & \omega_{h1}^- & 0 \\ 0 & 0 & \omega_{h2}^+ \end{pmatrix}, \tag{S10}$$

where $\omega_{h1}^{\pm} = \frac{1}{2}(\Delta_1 + \omega_{h2}^- \pm \sqrt{(\Delta_1 - \omega_{h2}^-)^2 + 4g_1^2})$.

In the case where $\Delta_2 \gg g_2$, the state with eigenfrequency $\omega_{h1}^- \approx 0$ is transmon like. The interaction between TLS1 and the transmon is unaffected by the presence of TLS2, bringing us back to the case of Eq. S7, with a single Rabi frequency of $\omega_{h1}^+ - \omega_{h1}^- \approx \sqrt{\Delta_1^2 + 4g_1^2}$. However, when $\Delta_2 \sim g_2$, the transmon and TLS2 become strongly hybridized, perturbing the TLS1-transmon interaction. Let's consider the SWAP spectroscopy experiment, where the excited-state transmon is flux-tuned close to the resonance frequency of TLS2. Due to the strong hybridization between TLS2 and the transmon, this experimental sequence effectively prepares a superposition of the states associated with eigenfrequencies ω_{h1}^- and ω_{h2}^+. Measurement of the transmon then reveals two Rabi frequencies for TLS1, one at $\omega_{h1}^+ - \omega_{h1}^-$ and another one at $\omega_{h1}^+ - \omega_{h2}^+$. This splits the original TLS1-transmon Rabi frequency curve of $\sqrt{\Delta_1^2 + 4g_1^2}$,

opening up a gap. The gap size is determined at $\Delta_2 = 0$, given by

$$\begin{aligned}&(\omega_{h1}^{+} - \omega_{h1}^{-}) - (\omega_{h1}^{+} - \omega_{h2}^{+})\\ =&\frac{1}{2}[\sqrt{(\Delta_1 + g_2)^2 + 4g_1^2} - (\Delta_1 - 3g_2)]\\ \approx& 2g_2 + \frac{g_1^2}{\Delta_1 + g_2},\end{aligned} \tag{S11}$$

where the last line in Eq. S11 takes the approximation $(\Delta_1 + g_2)^2 \gg 4g_1^2$.

We have highlighted such gaps in red boxes in Fig. S20. The flux biases at which these gaps occur correspond to resonance conditions with a second TLS. In our experiment, the flux biases coincide with the deviations from ideal chevron patterns in the time domain. Therefore it is likely that the imperfect patterns observed in the SWAP spectroscopy arise from the three-mode coupling of TLS-transmon-TLS, where a second TLS is involved. Importantly, it is worth noting, that rather than causing these spectral anomalies, the three-mode coupling could facilitate useful operations such as TLS-TLS entanglement[81].

G. Dependence of relaxation on temperature

1. *Temperature-dependent relaxation of transmon*

Before delving into possible TLS relaxation channels and presenting data on the temperature-dependent relaxation of TLS, we first visit the relaxation mechanisms for the transmon qubit. The understandings of these mechanisms are well-established. We employ a widely adopted model that accounts for relaxations from TLS/dielectric loss, quasiparticles (QP), and other contributions. Following ref.[4,44,45], the Q-factor of the transmon is determined by,

$$\frac{1}{Q} = \frac{1}{Q_{\text{TLS}}} + \frac{1}{Q_{\text{QP}}} + \frac{1}{Q_{\text{other}}}, \tag{S12}$$

where

$$Q_{\text{TLS}}(\bar{n}, T) = Q_{\text{TLS},0}\frac{\sqrt{1 + (\frac{\bar{n}^{\beta_2}}{DT^{\beta_1}})\tanh\left(\frac{\hbar\omega}{2k_BT}\right)}}{\tanh\left(\frac{\hbar\omega}{2k_BT}\right)}, \tag{S13}$$

and

$$Q_{\text{QP}} = Q_{\text{QP},0}\frac{e^{\Delta_0/k_BT}}{\sinh\left(\frac{\hbar\omega}{2k_BT}\right)K_0(\frac{\hbar\omega}{2k_BT})}. \tag{S14}$$

ω is the angular frequency of the transmon qubit; T the temperature; $\bar{n}$ the effective excited-state population; $Q_{\text{TLS},0}$, $Q_{\text{QP},0}$ the inverse of linear absorption due to TLS and quasiparticles; D, β_1, β_2 are parameters that characterize TLS saturation; Δ_0 the superconducting gap; K_0 the zeroth order modified Bessel function of the second kind.

This model gives a quantitative good fit, as shown in Fig. S21a in the gray dash-dotted line, that characterizes three distinct regimes as we increase the MXC plate temperature of the fridge: between 7–20 mK, transmon Q factor remains temperature-independent, described by Q_{other}; between 20–130 mK transmon Q increases slightly due to the saturation of TLS by thermal phonons; and above 130 mK, transmon Q decreases due to interactions with the thermally-activated QPs. We remark that the observed plateau in the measured Q-factor, that corresponds to the contribution of Q_{other}, is not necessarily interpreted as a truly temperature-independent loss channel. This plateau could potentially arise from a discrepancy between the true temperature of the transmon device and the temperature measured on the MXC plate, especially at the lowest MXC plate temperatures. At MXC plate temperatures below 20 mK, it is common for the true temperature of the transmon device to plateau at a higher temperature, typically around ~ 50 mK due to limited thermalization with the MXC plate[82]. When the MXC plate temperature is applied in Eq. S12 instead of the true device temperature, the effects from the plateaued transmon device temperature can manifest as a temperature-independent residual loss term Q_{other}. This phenomenon has been observed in various systems at milliKelvin temperatures, where the true device temperatures are estimated to plateau to a level around 50 mK[47].

2. Temperature-dependent relaxation of TLS

Now, we elaborate on the case study of TLS5 regarding its temperature-dependent relaxation, presented in Fig. 4b of the main text, as a prelude to the rich TLS physics enabled by the significantly extended TLS lifetime. In this experiment, we investigate the thermal bath by monitoring TLS5 as we warm up (WU) the mixing plate of the fridge from the base temperature of 7 mK to 193 mK, and cool down (CD) back to the base temperature, shown in Fig. S21a. No hysteresis is observed between the WU (red markers) and CD (yellow markers) paths. The quality factor Q of the TLS saturates to $Q \sim 2.5 \times 10^7$ at temperatures below ~ 75 mK, then drops by three orders of magnitude to $Q < 5 \times 10^4$ at 193 mK. Transmon Q is superimposed for reference, which agrees with a widely adopted model that considers effects from resonant TLS and quasiparticles[4,44,45], shown by the gray dash-dotted line, as discussed in the previous section, Eq. S12–S14. The TLS and transmon curves have inconsistent trends, suggesting that the temperature-dependent TLS relaxation is not dominated by the Purcell limit of the transmon.

For temperatures above 150 mK, the drop in TLS Q-factor seems to follow that of the transmon qubit, which is dominated by thermally-activated QPs. Here we assume a phenomenological QP loss-model given by:

$$Q_{\mathrm{TLS}}(T)^{-1} = \Gamma_{\mathrm{qp}} \frac{\sinh\left(\frac{\hbar\omega}{2k_{\mathrm{B}}T}\right) K_0\left(\frac{\hbar\omega}{2k_{\mathrm{B}}T}\right)}{e^{\Delta_0/k_{\mathrm{B}}T}}, \tag{S15}$$

where T is temperature, ω the TLS transition frequency, k_{B} the Boltzmann constant, $\Delta_0 = 1.764 \times 1.2$ K the superconducting gap of Al, and K_0 the 0-th order modified Bessel function of the second kind. This model describes quasiparticles (QP) in thermal equilibrium, which tunnel through the JJ and interact with the electric dipole of the TLS. The functional form resembles the thermal equilibrium QPs interacting with SC qubits[44], as will be discussed in Sec. II H 4, and in ref.[50]. This loss channel could result in the many orders-of-magnitude Q-factor change within a small temperature range of 200 mK.

We fix the superconducting gap of Al Δ_0, which leaves only one free parameter, Γ_{qp}, in the model. Using the mixing plate temperature for the model, Eq. S15 yields the gray dashed line in Fig. S21a. As can be seen, predictions of this simple model diverges from experimental data at temperatures below 150 mK. However, recent studies[47–49] show an excess of non-equilibrium (ne) QP population, which corresponds equivalently to thermal equilibrium QP population at approximately 150 mK. Comparing the measured Q-factor to the gray dashed line, we map each mixing plate temperature T_{MXC} to an effective temperature T_{eff}, shown in Fig. S21b. T_{eff} is empirically fitted, using $T_{\mathrm{eff}}(T_{\mathrm{MXC}}) = A\sqrt{1 + B\tanh(C/T_{\mathrm{MXC}})}/\tanh(C/T_{\mathrm{MXC}})$, represented by the blue solid line in Fig. S21b. Using the effective temperature $T_{\mathrm{eff}}(T_{\mathrm{MXC}})$ in Eq. S15, yields the black solid line in Fig. S21a, in qualitative agreement with measured data. The transmon fit using T_{eff} remains largely unaffected. If QPs were to explain the plateauing behavior in TLS Q-factor, this analysis predicts a QP saturation temperature of approximately 130 mK. This is consistent with recent studies[47–49] of ne QP population in Al superconducting circuits, which infer a QP population with effective temperature of 120-150 mK.

We emphasize that TLS-QP coupling is simply one possible explanation for the observed temperature-dependent TLS relaxation behavior. This behavior deviates from predictions of the standard tunneling model of TLS[22], and reveals previously unexplored TLS physics that requires further investigation. Other possible mechanisms that could contribute to the plateauing behavior in the measured TLS Q includes temperature-independent channels such as TLS coupling to heavily damped grain-boundary motion in the polycrystalline Al layers[10,46]. In either case, this temperature-dependent TLS Q behavior deviates from predictions of the standard tunneling model of TLS[22]. It reveals previously unexplored TLS physics that requires further investigation.

Regarding the temperature-dependent measurements of TLS, it is important to note that at elevated temperatures, we observed not only a decrease in signal-to-noise ratio, but also fluctuations in the T_1 relaxation curve of the TLS when we repeat the measurements. These fluctuations are likely attributed to reconfigurations of the relaxation bath. To mitigate this issue, we averaged over many T_1 relaxation curves in our experiment. Subsequently, all the long averaged relaxation curves were fitted to a stretched exponential model, given by

$$p(t) = A\exp[-(t/T_1)^n] + B. \tag{S16}$$

This averaging and fitting approach was applied to TLS5 data in Fig. S21. The distribution of fitted exponent yields $n_{\mathrm{TLS5}} = 0.77 \pm 0.14$.

H. Possible relaxation channels for TLS

Guided by the measurements of temperature-dependent relaxation for both TLS and the transmon, we will now discuss in more detail the possible relaxation channels for the TLS. In this context, we will outline prevalent relaxation

mechanisms expected in our system, evaluate their alignment with the experimental data, and where applicable, suggest experiments or simulations for further investigation. In the subsequent sections, we will first discuss three potential origins of the temperature-independent loss of TLS at the lowest temperatures. Subsequently, our focus shifts towards possible contributions to the thermally-activated TLS relaxation, exploring relaxation mechanisms due to TLS, phonons, and QPs.

1. *Temperature-independent loss channels*

First, as discussed above in Sec. II G 2, a possible channel comes from the ne QPs interacting with TLS, causing energy relaxation of TLS. These ne QPs are thought to originate from high-energy incidents, such as cosmic μ rays and background γ ray radiation, which would hit the microchip. These energetic events break Cooper-pairs and excite high-energy phonons that could propagate through the entire chip. Along the way, these high-energy phonons would dissipate energy, leading to the generation of ne QPs[47,83–85]. This results in ne QPs of population much higher than expected for equilibrium QPs at the temperature of the microchip, resulting in an effective QP temperature at approximately 150 mK[47–49]. Unfortunately it is challenging to filter out these ne QPs. Unlike their equilibrium counterparts, the population of ne QPs arising from high-energy events, and correspondingly their contribution to TLS relaxation, remains independent of temperature.

Similarly, at the lowest temperatures, the true device temperature could be higher than the MXC plate temperature, due to insufficient thermalization between the device and the MXC plate, as discussed for the transmon device in Sec. II G 1. The same argument can be extended to TLS as well. In this case, TLS might experience an even higher temperature plateau than the transmon device. This is primarily attributed to their poorer thermalization, due to factors such as their smaller size, longer lifetime, and a suppressed thermal bath (of phonons). The saturation in temperature then manifest as a temperature-independent residual loss channel. Finally, there is a temperature-independent mechanical loss channel stemming from the viscous behavior of grain-boundaries in the polycrystalline aluminum[10,46]. Although we have focused on the QP loss-model (Eq. S15), and the associated higher effective temperature due to the temperature-independent population of ne QPs to explain our observation in Fig. S21, the other two temperature-independent loss channels discussed here, and potentially other relaxation channels that we have not considered, should still be considered possibilities.

2. *Temperature-dependent loss channels: other TLS*

Turning our attention to the temperature-dependent loss pathways, we first explore resonant interactions between a given TLS and other nearby TLS, all with their frequencies lying inside the acoustic bandgap. In Sec. II B, we have obtained a TLS density of $\sigma = 0.6$ GHz$^{-1}\mu$m^{-2} in the AlO$_x$ barrier layer. Extending this TLS density estimation to the Al and Si surfaces, we expect on average a total of 120 TLS per GHz for a 10 μm$\times$10 μm region. This accounts for 60 TLS on both the top and bottom side of the 220 nm Si device layer. The chosen area of 10 μm$\times$10 μm approximates the size of the acoustic metamaterials and the enclosed JJ region. We make the assumption that TLS outside the JJ maintain comparable properties to those within the JJ due to the presence of the same acoustic bandgap metamaterial. This assumption gives the TLS relaxation time of $T_1 \sim 500$ μs, and the coherence time of $T_2^* \sim 1$ μs in this extended region. Importantly, the spectral linewidths of these TLS are dominated by dephasing, resulting in a linewidth of ~ 1 MHz. Given the presence of 120 such TLS over a 1 GHz span, the average detuning between neighboring TLS frequencies is ~ 8 MHz, which significantly exceeds the linewidth. As a result, it is reasonable to infer that resonant interactions among TLS are unlikely to contribute significantly to TLS relaxation.

Continuing our exploration of TLS-TLS interactions, we extend our discussions to off-resonant interactions between TLS. Our preliminary assessment leads us to conclude that off-resonant TLS-TLS interactions do not account for the plateau of TLS Q-factor at the lowest temperatures. As will be discussed in Sec. II H 3, the temperature scaling of the relaxation rate arises from the frequency scaling of the thermal bath modes in the relaxation rate expression. This frequency scaling, in turn, comes from the DOS of the bath modes as well as the frequency-dependence of the system-bath interactions (the transition matrix element). Previous studies of TLS have shown that TLS DOS is either independent of frequency, or has a weak frequency-dependence $\sim \omega^\mu$, with $\mu \simeq 0.3$[3,22,86,87]. The interaction between TLS is dipolar, determined only by their dipole magnitude, orientation, and relative position, which are all frequency-independent. Consequently, the relaxation rate due to off-resonant TLS will have at most a weak power-law temperature scaling of T^μ. This mismatches the strong temperature scaling we have observed for $T \gtrsim 50$ mK.

The weak temperature scaling from a bath of off-resonant TLS, however, necessitates an evaluation of their potential contribution to the plateau of TLS Q-factor at the lowest temperatures. Given a TLS density of $\sigma = 0.6$ GHz$^{-1}\mu$m^{-2}, the average distance between TLS is $1/2\sqrt{\sigma} = 0.65$ μm, corresponding to a TLS-TLS coupling strength of $g = 5$ kHz,

when the two dipoles are aligned. The average detuning between TLS with adjacent frequencies is $\Delta \sim 250$ MHz. The linewidth for the bath TLS is assumed around $\Gamma_{2,\text{bath}} \sim 1$ MHz. Using similar approximations that will be discussed next in Sec. II H 3, we reach at the relaxation rate attributed to the interaction with a singular off-resonant bath TLS

$$\Gamma_{1,\text{TLS}} \approx \frac{g^2 \Gamma_{2,\text{bath}}}{\Delta^2 + \Gamma_{2,\text{bath}}^2} = 0.4 \text{ mHz}. \tag{S17}$$

This contribution is seven orders of magnitude weaker than the measured relaxation rate of approximately ~ 2 kHz. Further numerical simulation corroborates that with the TLS density of $\sigma = 0.6$ GHz$^{-1}\mu$m^{-2} and a linewidth of $\Gamma_{2,\text{bath}} \sim 1$ MHz, interactions between TLS contribute negligibly to the Q-factor plateau at the lowest temperatures for the central TLS.

3. *Temperature-dependent loss channels: Phonons outside the acoustic bandgap*

In this section, we explore TLS relaxation due to its interaction with phonons. We consider a single phonon process, in which the TLS relaxes by emitting a single phonon. For this discussion, we draw upon the key results and notations from ref.[13], where a more detailed analysis is provided. The relevant Hamiltonian between the TLS and a stress wave associated with the phonon mode s is

$$\hat{\mathcal{H}}_{\text{TLS}-s} = \frac{\omega_{\text{TLS}}}{2}\hat{\sigma}_z + \omega_s(\hat{b}_s^\dagger \hat{b}_s + \frac{1}{2}) + (g_{t,s}\hat{\sigma}_x + g_{l,s}\hat{\sigma}_z)(\hat{b}_s + \hat{b}_s^\dagger), \tag{S18}$$

where ω_{TLS} and ω_s are the frequencies of the TLS and the phonon mode s, $g_{t,s}$, $g_{l,s}$ their transverse and longitudinal coupling, $\hat{\sigma}$ the Pauli operator for TLS, and $\hat{b}^\dagger(\hat{b})$ the creation (annihilation) operator for phonon mode s.

In the case of resonant decay from TLS into the phonon bath, the $g_t\hat{\sigma}_x$ term in Eq. S18 dominates. In this context, neglecting pure dephasing of the phonons, we arrive at the relaxation rate of TLS induced by phonon mode s, approximated by

$$(\delta\Gamma_{1,\text{TLS}})_s \approx \frac{g_{t,s}^2 \gamma_s (2n_s + 1)}{(\omega_{\text{TLS}} - \omega_s)^2 + (\gamma_s/2)^2}. \tag{S19}$$

Considering a phonon bath in thermal equilibrium, characterized by the Bose-Einstein distribution, where $2n_s + 1 = \coth[\hbar\omega_s/2k_BT]$, Eq. S19 leads to[13]

$$(\delta\Gamma_{1,\text{TLS}})_{ph} \approx \sum_s [\frac{g_{t,s}^2 \gamma_s}{(\omega_{\text{TLS}} - \omega_s)^2 + (\gamma_s/2)^2}] \coth[\hbar\omega_s/2k_BT]. \tag{S20}$$

In the limit of a continuum phonon bath, the summation in Eq. S20 is replaced by an integral. The primary contribution to the integral comes from the integration range $\omega_s \in [\omega_{\text{TLS}} - \gamma_s/2, \omega_{\text{TLS}} + \gamma_s/2]$, yielding

$$(\delta\Gamma_{1,\text{TLS}})_{ph,cont.} \approx 4\rho_{ph}[\omega_{\text{TLS}}] g_{t,s}^2 \coth[\hbar\omega_s/2k_BT], \tag{S21}$$

where $\rho_{ph}[\omega]$ is the phonon DOS at frequency ω. For Debye model phonons in D-dimensions, $\rho_{ph}[\omega] \propto \omega^{\text{D}-1}$, and $g_{t,s} \propto \sqrt{\omega_s}$ from the vacuum strain field amplitude. This leads to $(\delta\Gamma_{1,\text{TLS}})_{ph,cont.} \propto \omega_{\text{TLS}}^{\text{D}}$. We will show in the following that the frequency scaling yields the same scaling for temperature.

We now consider the reverse process—a phonon decays into the TLS bath. In particular, we skip the discussion of resonant decay (dominated by the $g_t\hat{\sigma}_x$ term), and instead focus on the off-resonant 'relaxation' process, which is dominated by the $g_l\hat{\sigma}_z$ term in Eq. S18. The $\hat{\sigma}_z$ interaction shifts the frequencies of phonons, displacing them away from thermal equilibrium. Through a higher-order process between the TLS and the phonon modes, the TLS draws energy from the phonon modes, giving rise to the 'relaxation' process of phonons. Integrating the contributions from all TLS, and assuming a T_1 limited T_2 for the phonon mode s, yield[13]

$$(\delta\gamma_s)_{\text{rel}} \approx \sum_{\text{TLS}} (\frac{2g_{l,s}^2}{\omega_s})(\frac{\hbar\Gamma_{1,\text{TLS}}}{k_BT}) \operatorname{sech}^2[\hbar\omega_{\text{TLS}}/2k_BT]. \tag{S22}$$

Assuming the energy damping of TLS in the TLS bath is dominated by resonant decay into phonon modes, $\Gamma_{1,\mathrm{TLS}} = (\delta\Gamma_{1,\mathrm{TLS}})_{ph,cont.}$, we can plug in Eq. S21 into Eq. S22, which leads to the scaling

$$\begin{aligned}(\delta\gamma_m)_{\mathrm{rel}} \propto & \sum_{\mathrm{TLS}} \frac{\omega_{\mathrm{TLS}}^{\mathrm{D}}}{k_B T} \coth[\frac{\hbar\omega_{\mathrm{TLS}}}{2k_B T}] \,\mathrm{sech}^2[\frac{\hbar\omega_{\mathrm{TLS}}}{2k_B T}] \\ \approx & \int_0^\infty \frac{\omega_{\mathrm{TLS}}^{\mathrm{D}}}{k_B T} \coth[\frac{\hbar\omega_{\mathrm{TLS}}}{2k_B T}] \,\mathrm{sech}^2[\frac{\hbar\omega_{\mathrm{TLS}}}{2k_B T}] \rho_{\mathrm{TLS}} d\omega_{\mathrm{TLS}} \\ = & 2\rho_{\mathrm{TLS}} (\frac{k_B T}{\hbar})^{\mathrm{D}} \int_0^\infty x^{\mathrm{D}} \,\mathrm{csch}[x] dx.\end{aligned} \tag{S23}$$

In the last line, we used the identity $\mathrm{sech}^2[\hbar\omega/2k_BT]\coth[\hbar\omega/2k_BT] = 2\,\mathrm{csch}[\hbar\omega/k_BT]$, and made the assumption of a frequency-independent TLS density ρ_{TLS}. As promised earlier, the frequency scaling $\propto \omega^{\mathrm{D}}$ in Eq. S21 leads to an equivalent temperature scaling $\propto T^{\mathrm{D}}$ in Eq. S23, which directly reflects the dimension D of the system. More generally, the temperature scaling of off-resonant relaxation processes is a result of integrating over the frequency-dependent terms in the relaxation rate. This relation has been observed across a wide variety of examples, such as TLS-phonon interaction[13], two-phonon Orbach-like process[88], spin-phonon relaxation[89], and three-phonon scattering[90].

Taking the example of the TLS-phonon interaction, the frequency scaling of phonon relaxation discussed above involves two contributions, an $\omega^{\mathrm{D}-1}$ term from the phonon DOS, and an ω term from the square of TLS-phonon coupling strength. We argue that for the long-lived TLS, one expects a similar scaling for the single phonon, off-resonant 'relaxation' process. In our case, the effective system dimension for thermally activated phonons is $\mathrm{D} = 2$ at lower temperatures, and it increases to $\mathrm{D} > 2$ at higher temperatures, when high frequency phonons that see a semi-3D DOS start to be thermally populated. The predicted T^2 dependence, however, does not align with our experimental data in Fig. S21. Specifically, the T^2 dependence is too strong to account for the almost temperature-independent relaxation at $T \lesssim 50$ mK, and too weak to explain the observed rapid decrease in T_1 at $T \gtrsim 50$ mK.

Additionally, numerical modelling of the damping of TLS within the acoustic bandgap due to quasi-phonon modes for a similar acoustic structure was performed in ref.[13], indicating that TLS damping of this nature would limit T_1 to 100 ms or more, two-orders-of-magnitude greater than measured here. We also don't observe evidence of an increasing trend of the low-temperature T_1 of TLS as their frequencies move deeper into the acoustic bandgap, which one would expect if this was the source of the T_1 limit.

4. Temperature-dependent loss channels: Quasiparticles

For the discussions of quasiparticles (QP), we first make the distinction between the contributions of QPs in thermal equilibrium (eq), and those in non-equilibrium (ne). The ne QPs are generated by bursts of high-energy events[47]. Notably, these high-energy events, and correspondingly the distribution of ne QP, are temperature-independent, and are already discussed in Sec. II H 1. The temperature-dependent contribution comes exclusively from the eq QPs. For the eq part, we follow the treatment of ref.[44,91], and list the key steps here for readers' convenience.

The QP interacts with the qubit by tunneling through the Josephson Junction, resulting in the system Hamiltonian

$$\mathcal{H} = \mathcal{H}_\phi + \mathcal{H}_{\mathrm{qp}} + \mathcal{H}_T. \tag{S24}$$

The first term is the SC qubit Hamiltonian, which for transmon is

$$\mathcal{H}_\phi = 4E_C\hat{n}^2 - E_J\cos\hat{\phi}. \tag{S25}$$

The second term is the BCS Hamiltonian for the QPs,

$$\mathcal{H}_{\mathrm{qp}} = \sum_{j=L,R} \mathcal{H}_{\mathrm{qp}}^j,\ \mathcal{H}_{\mathrm{qp}}^j = \sum_{n,\sigma} \epsilon_n^j \alpha_{n\sigma}^{j\dagger}\alpha_{n\sigma}^j, \tag{S26}$$

where $\alpha_{n\sigma}^j(\alpha_{n\sigma}^{j\dagger})$ are the annihilation (creation) operators for quasiparticle in the lead j that has spin $\sigma =\uparrow,\downarrow$, and energy ϵ_n in the single-particle energy level n. The last term describes QP tunneling through the junction, and under simplifications relevant to superconducting circuits,

$$\mathcal{H}_T = i\tilde{t}\sum_{n,m,\sigma} \sin\frac{\hat{\phi}}{2}\alpha_{n\sigma}^{L\dagger}\alpha_{m\sigma}^R + \mathrm{h.c.}, \tag{S27}$$

where $\tilde{t}$ is the electron tunneling amplitude.

When calculating the QP impact on the transmon qubit, Fermi's golden rule gives

$$\begin{aligned}\Gamma_{i\to f} =& 2\pi \sum_{\{\lambda_{\rm qp}\}} |\langle f, \{\lambda_{\rm qp}\}|\mathcal{H}_T|i, \{\eta_{\rm qp}\}\rangle|^2 \\ &\times \delta(E_{\lambda,qp} - E_{\eta,qp} - \omega_{if}),\end{aligned} \tag{S28}$$

where $E_{\eta,qp}(E_{\lambda,qp})$ is the energy of the QP in its initial (final) state $\{\eta_{\rm qp}\}$ ($\{\lambda_{\rm qp}\}$), ω_{if} is the energy difference of the qubit in the initial and final state. An average over the initial quasiparticle state following its distribution is taken, which is inexplicit in the equation.

In the low energy regime, it has been shown that the qubit dynamics and QP kinetics are separable[91], yielding

$$\Gamma_{i\to f} = 2\pi |\langle f| \sin\frac{\hat{\phi}}{2}|i\rangle|^2 S_{\rm qp}(\omega_{if}). \tag{S29}$$

This is an established model describing the QP interaction with superconducting qubit. Eq. S29 takes the form of Fermi's golden rule, where $S_{\rm qp}(\omega_{if})$ represents the current spectral density of quasiparticles tunneling through the JJ, that interacts with the phase degrees of freedom of the transmon qubit through the matrix element of $\langle f|\sin\frac{\hat{\phi}}{2}|i\rangle$.

Within a similar context, we consider the interaction between QPs and TLS inside the JJ, in a regime where the TLS dynamics and QP kinematics are separable. The matrix element coupling the electric dipole of the TLS to the electric current formed by QPs tunneling through the junction depends on the details of the microscopic configuration. Here we skip discussions on those details and denote it as A_{if}. The current spectral density of QPs tunneling through the junction $S_{\rm qp}(\omega)$ stays the same as in the previous discussion,

$$\begin{aligned}S_{\rm qp}(\omega) =& \frac{16E_J}{\pi}\int_0^\infty dx \frac{1}{\sqrt{x}\sqrt{x+\omega/\Delta}} f_E[(1+x)\Delta] \\ &\times \{1 - f_E[(1+x)\Delta + \omega]\}),\end{aligned} \tag{S30}$$

where f_E is the distribution function, Δ the gap parameter.

In thermal equilibrium (assuming Boltzmann distribution), and at low temperatures $T \ll \Delta$, Eq. S30 undergoes further simplification,

$$S^{eq}_{\rm qp}(\omega) = \frac{16E_J}{\pi} e^{-\Delta/T} e^{\omega/2T} K_0(\frac{|\omega|}{2T}). \tag{S31}$$

Using the relation

$$S^{eq}_{\rm qp}(-\omega)/S^{eq}_{\rm qp}(\omega) = e^{-\omega/T}, \tag{S32}$$

we find the total TLS relaxation rate due to QPs

$$\begin{aligned}\Gamma_{\rm qp} &= 2\pi|A_{if}|^2[S^{eq}_{\rm qp}(\omega) - S^{eq}_{\rm qp}(-\omega)] \\ &= 2\pi|\tilde{A}_{if}|^2 e^{-\Delta/T}\sinh\Big(\frac{\omega}{2T}\Big)K_0(\frac{|\omega|}{2T}).\end{aligned} \tag{S33}$$

It's worth noting that the functional form of this expression is the same as that of the transmon-QP relaxation in Eq. S14. The matrix element term $\tilde{A}_{if}$ describes the coupling between eq QP current and the electric dipole of TLS, which depends on the details of the microscopic configuration, that is beyond the scope of this work.

The consideration of TLS-QP interaction naturally arises for TLS located inside the JJ. In this scenario, the current of QPs tunneling through the JJ interacts with the electric dipole of TLS. Eq. S33 qualitatively describes the temperature-dependent TLS relaxation well at high temperatures $T \gtrsim 150$ mK, as shown in Fig. S21. We therefore attribute eq QPs as one of the relevant TLS relaxation channels.

In our experiment, the prominence of the QP contribution emerges from our selection of TLS located inside the JJ. This specific configuration results in a strong coupling between the TLS and QPs tunneling through the JJ, due to the close proximity. We note that the QP current spectral density, and correspondingly the QP-TLS interaction, is expected to vary significantly across different regions of the device. An example is the substrate-air interface, where QPs are altogether absent.

We remark that all the aluminum films used in our devices are of similar thicknesses (30 nm and 50 nm for the bottom and top electrodes of the JJ, and 80 nm for the ground plane). This puts our device in a regime where

the expected difference in the superconducting gap between the two electrodes, and between the electrodes and the ground layer, are well below the qubit frequency. Therefore QPs are relatively free to diffuse into the JJ region and tunnel through, causing energy relaxation of both the transmon qubit (and possibly of the TLS). In this context, we expect superconducting gap engineering, as recently demonstrated for transmon qubits[64], will also work effectively for suppressing (potential) QP damping of TLS in the junction. This could lead to significant (x1000) increases in TLS T_1 lifetime at temperatures below approximately 130 mK beyond that measured in this work. Such a future experiment could also be used to validate or rule out the proposed model of TLS-QP interaction in the junction.

I. Direct control of TLS

1. *TLS pulses*

In this section, we describe the technique of directly controlling a TLS. This is achieved by sending a strong microwave pulse resonating with the TLS down the XY line of the transmon qubit. The direct control of TLS is possible due to the mutual interaction and the resulting hybridization between the transmon qubit (q) and TLS, governed by the Hamiltonian

$$\begin{aligned}\mathcal{H} &= \frac{\omega_q}{2}\hat{\sigma}_q^z + \frac{\omega_{\mathrm{TLS}}}{2}\hat{\sigma}_{\mathrm{TLS}}^z + \hat{H}_{\mathrm{int}},\\ \mathcal{H}_{\mathrm{int}} &= g(\hat{\sigma}_q^+\hat{\sigma}_{\mathrm{TLS}}^- + \hat{\sigma}_q^-\hat{\sigma}_{\mathrm{TLS}}^+),\end{aligned} \tag{S34}$$

where ω denote their frequencies, $\hat{\sigma}^z, \hat{\sigma}^\pm$ are the Pauli operators.
The interaction term $\mathcal{H}_{\mathrm{int}}$ hybridizes the transmon and the TLS. The hybridization, even at the presence of a detuning between the transmon and the TLS, gives the TLS-like eigenstate a little transmon character, that enhances TLS' coupling with the transmon's XY line. This technique has been used in the context of directly controlling TLS in a phase qubit[41], in the cross-resonance gate between two coupled superconducting qubits[92,93], as well as in accelerating nuclear spin gates in quantum registers in diamond[94].

An example is shown in Fig. S22. Here, a Rabi pulse of varying duration and microwave frequency drives the state of the TLS, which is subsequently read out through the transmon qubit. The resultant Rabi chevron pattern, notably of the TLS under direct control, demonstrates the feasibility and precision in controlling TLS using this technique. In this specific experiment, the interaction strength and detuning between the TLS and the transmon qubit are $g = 47.7$ MHz, $\Delta = 1.7$ GHz. The driving power of the XY pulse is approximately ~ 17 dB stronger than what is typically used for controlling the transmon qubit.

2. *TLS relaxation time with TLS pulses*

The technique of direct TLS control, as discussed above, allows us to calibrate pulses for the TLS and prepare the TLS in its excited-state independently from the transmon qubit. However, it's important to note that the readout process still involves the transmon qubit. In Fig. S23, we present the T_1 energy relaxation curve of TLS31, showcasing a comparison between the two methods: preparing the initial TLS excited-state with a TLS π pulse (direct drive, blue triangles) and through swapping the excitation from the transmon qubit (SWAP, red squares).

The two distinct methods of preparing the TLS in its excited-state yield different relaxation curves. Notably, the direct drive method gives a shorter $T_1 = 129 \pm 5$ μs, whereas the SWAP method gives a longer $T_1 = 177 \pm 10$ μs. This discrepancy can be attributed to the high power of the microwave pulse required for the direct drive, which is approximately ~ 17 dB stronger than that used for the transmon qubit. The high power microwave can generate QPs[76,77] which subsequently accelerate the relaxation of TLS through the mechanism discussed in Sec. II H 4. Following this observation from the first cool-down (CD1), we have been using the SWAP method exclusively for the preparation of TLS excited-states in the following TLS T_1 measurements.

Throughout the measurements, particularly during CD1, a notable portion of TLS were characterized by the direct drive method, as indicated by the † symbol in Table S3 and Table S4. We argue that for TLS with resonant frequency lying outside the acoustic bandgap, the relaxation rate induced by the QPs originating from high-power microwave pulses, is likely minor when compared to other relaxation mechanisms (e.g. spontaneous phonon emissions). Revisiting the case of TLS31, we compute the relaxation rate due to microwave induced QPs $\tau_{\mathrm{QP}}^{-1} = 1/129\ \mu\mathrm{s} - 1/177\ \mu\mathrm{s} = 476^{-1}\ \mu\mathrm{s}^{-1}$. This value is two orders of magnitude smaller than the average relaxation rate of $4^{-1}\ \mu\mathrm{s}^{-1}$ for TLS outside the bandgap, contributing negligibly to their relaxation. While for TLS inside the bandgap, these QPs generated by the high-power microwave pulses could potentially lower the measured T_1 notably, as shown in Fig. S23. As a result,

the reported ratio between the T_1 values of TLS frequencies inside vs outside the acoustic bandgap represents an *underestimation* of the impact from acoustic metamaterials.

3. TLS relaxation curve with TLS pulses

Another phenomenon emerges when measuring TLS relaxation using the direct drive method. In certain cases, we have observed deviations of the TLS T_1 relaxation curve from a simple exponential decay. An illustrative example from TLS36 is presented in Fig. S24. To analyze the data, we fit it with a simple exponential decay curve,

$$p(t) = A\exp(-t/T_1) + B, \tag{S35}$$

indicated by the red solid line, as well as a double exponential decay curve[48,95],

$$p(t) = Ae^{\langle n_{\mathrm{qp}}\rangle(\exp[-t/T_{1,\mathrm{qp}}]-1)}e^{-t/T_1} + B, \tag{S36}$$

indicated by the blue solid line. Eq. S36 was introduced in refs.[48,95] for superconducting qubits, to disentangle the relaxation rate induced by quasiparticles from other relaxation channels. Here, $\langle n_{\mathrm{qp}}\rangle$ is the average quasiparticle population, $T_{1,\mathrm{qp}}$ is the relaxation time due to one quasiparticle, and T_1 is the relaxation time from other decay channels.

In experiments where the TLS relaxation curve no longer adheres to a simple exponential decay, as observed in Fig. S24, the double exponential fit is adopted. Consequently, we report T_1 from Eq. S36 in these cases. As previously discussed in Sec. II H 4, we propose that the relaxation of TLS inside the JJ, induced by interaction with QPs, follows the same functional form as the interaction between the transmon qubit and QPs. This similarity justifies the application of Eq. S36 for characterizing TLS T_1 relaxation with an explicit contribution from QP. We note that the direct TLS drive method does not always lead to double exponential decays. A case of simple exponential decay under direct TLS drive can be found in Fig. S23. In these cases, we fit the measured TLS relaxation curve to a simple exponential decay model, and report the corresponding underestimate of fitted T_1 values.

Device	$\omega/2\pi$ (GHz)	α (MHz)	$\omega_{\rm RR}/2\pi$ (GHz)	g (MHz)	T_1 (μs)
Chip-A Q_1	6.48	-182.5	7.26	74.0	4.5
Chip-A Q_2	6.29	-166.7	7.13	71.6	2.1
Chip-A Q_3	6.11	-159.3	7.00	66.8	3.0
Chip-A Q_4	5.98	-152.6	6.88	67.5	3.2
Chip-B Q_1	5.77	-182.1	6.22	71.8	1.5
Chip-B Q_2	5.57	-171.3	6.11	71.0	6.0
Chip-B Q_3	5.44	-162.7	6.01	70.1	3.5

TABLE S1. **Transmon qubit parameters.** Chip-A is designed to resolve the upper edge of the acoustic bandgap. This chip hosts four qubits, $Q_{1–4}$, with upper sweet spot frequencies above the upper edge of the acoustic bandgap, simulated to be around 5.814 GHz. As for Chip-B, designed to resolve the lower edge of the acoustic bandgap, three fully functional qubits, $Q_{1–3}$, have upper sweet spot frequencies above the lower edge of the acoustic bandgap, simulated to be around 4.442 GHz. Additional qubit parameters are provided in the table, including the qubit's anharmonicity α, the frequency of its corresponding readout resonator $\omega_{\rm RR}$, the coupling strength g to the resonator, and the typical T_1 relaxation time measured at the sweet spot. We note that these values are representative, subject to slight variations after each thermal cycling.

Al thickness (nm)	f_1 (GHz)	f_2 (GHz)
0	**4.442**	6.033
30	4.417	5.979
50	4.389	**5.814**

TABLE S2. **Simulated acoustic bandgaps for three different unit cells.** The tabulated information outlines the acoustic bandgap frequencies for the three unit cell types, as illustrated in the blue, red, and green dashed boxes in Fig. S1b. The corresponding band structures are plotted in Fig. S3. The table's columns specify the thickness (th.) of Al leads from the JJ passing through the cross-shield unit cell, as well as the respective lower and upper edges of the acoustic bandgap (f_1 and f_2). The bandgap for the entire structure, given by the frequency overlap between these three unit cell types, spans from 4.442 GHz to 5.814 GHz, highlighted in bold.

TLS index	freq. (GHz)	g (MHz)	T_1 (μs)	host qubit
1	6.3935	9.4	$1.9 \pm 0.5^{\dagger}$	Q_1 CD1
2	5.8818	7.4	$7.1 \pm 0.6^{\dagger}$	Q_1 CD1
3	5.2063	21.1	$283 \pm 51^{\dagger}$	Q_1 CD1
4	5.0730	22.3	$1611 \pm 188^{\dagger}$	Q_1 CD1
5	5.8996	7.2	$0.49 \pm 0.02^{\dagger}$	Q_1 CD1
6	5.7980	20.3	$35 \pm 5^{\dagger}$	Q_1 CD1
7	6.1647	$3.7^{\$}$	$199 \pm 27^{\dagger}$	Q_1 CD1
8	5.4359	19.6	$948 \pm 223^{\dagger}$	Q_1 CD1
9	6.1819	$3.4^{\$}$	2.7 ± 0.3	Q_1 CD2
10	6.0677	26.6	1.87 ± 0.16	Q_1 CD2
11	5.9024	16.2	1.90 ± 0.12	Q_1 CD2
12	5.7953	6.9	215 ± 15	Q_1 CD2
13	5.6563	21.7	1116 ± 203	Q_1 CD2
14	6.2740	$4.5^{\$}$	7.2 ± 1.6	Q_2 CD1
15	5.6891	$4.8^{\$}$	$2726 \pm 1026^{\dagger}$	Q_2 CD1
16	5.6534	9.7	$544 \pm 131^{\dagger}$	Q_2 CD1
17	4.9745	15.3	$25 \pm 3^{\dagger}$	Q_2 CD1
18	6.0877	30.2	13.2 ± 3.9	Q_3 CD1
19	5.9581	24.9	10.7 ± 1.5	Q_3 CD1
20	5.7359	$3.8^{\$}$	3.6 ± 0.3	Q_3 CD1
21	5.4867	10.2	$135 \pm 85^{\dagger}$	Q_3 CD1
22	4.8196	$4.2^{\$}$	5424 ± 830	Q_3 CD1
23	4.6952	8.1	90 ± 38	Q_3 CD1
24	5.2905	9.4	524 ± 74	Q_3 CD2
25	4.6925	6.9	571 ± 77	Q_3 CD2
26	4.5098	15.7	5.6 ± 0.8	Q_3 CD2
27	4.4604	-	3.8 ± 0.7	Q_3 CD2
28	5.4069	-	$451 \pm 82^{\dagger}$	Q_4 CD1
29	5.4097	-	$830 \pm 185^{\dagger}$	Q_4 CD1
30	5.2404	7.3	$178 \pm 98^{\dagger}$	Q_4 CD1
31	5.1759	47.7	177 ± 10	Q_4 CD1
32	5.8521	11.3	18 ± 2	Q_4 CD2
33	4.9567	10.4	$893 \pm 289^{\dagger}$	Q_4 CD2
34	4.3428	6.4	$4.5 \pm 0.5^{\dagger}$	Q_4 CD2

TABLE S3. **List of TLS parameters measured on Chip-A.** The provided list compiles the parameters obtained from characterizing 34 TLS using the four transmon qubit devices on Chip-A. The parameters listed include TLS frequency, interaction strength g with the transmon qubit, as well as their T_1 relaxation times, with one standard deviation uncertainty quoted. The hosting transmon qubit device for the TLS and the specific cool-down cycle when these TLS were characterized, are specified at the end of the list. †: Measurements conducted using the direct TLS control method (Sec. III 1), which likely results in shorter TLS T_1 measurements due to microwave generated quasiparticles. $ or -: Signify cases where the coupling strength g could not be extracted with high confidence. This might arise from small g values, or overlapping TLS avoided crossings, as identified in the SWAP spectroscopy. CD1/CD2: Indicate the cool-down cycle during which the TLS were measured.

TLS index	freq. (GHz)	g (MHz)	T_1 (μs)	host qubit
35	5.6481	11.7	$260 \pm 21^{\dagger}$	Q_1 CD1
36	4.9813	9.2	$1105 \pm 458^{\dagger}$	Q_1 CD1
37	4.7006	11.3	$85 \pm 8^{\dagger}$	Q_1 CD1
38	4.4365	28.4	$0.25 \pm 0.02^{\dagger}$	Q_1 CD1
39	5.2866	$4.8^{\$}$	$257 \pm 46^{\dagger}$	Q_1 CD1
40	4.8542	-	255 ± 24	Q_1 CD2
41	4.7279	19.6	287 ± 33	Q_1 CD2
42	4.5474	11.7	478 ± 39	Q_1 CD2
43	4.3888	7.5	2.6 ± 0.5	Q_1 CD2
44	4.2304	-	1.4 ± 0.1	Q_1 CD2
45	3.6385	30.2	3.2 ± 0.3	Q_1 CD2
46	5.2956	10.9	474 ± 298	Q_2 CD1
47	4.4225	12.1	4.0 ± 0.3	Q_2 CD1
48	4.0957	23.2	4.3 ± 0.6	Q_2 CD1
49	4.8908	3.9	505 ± 91	Q_2 CD2
50	4.3205	27.4	0.71 ± 0.02	Q_2 CD2
51	4.0277	10.3	101 ± 11	Q_2 CD2
52	5.1151	3.0	652 ± 103	Q_3 CD2
53	4.9870	9.2	866 ± 116	Q_3 CD2
54	4.3282	9.2	4.6 ± 1.6	Q_3 CD2
55	3.7421	-	11.5 ± 2.9	Q_3 CD2
56	3.7567	-	22.1 ± 1.0	Q_3 CD2

TABLE S4. **List of TLS parameters measured on Chip-B.** The provided list compiles the parameters obtained from characterizing 22 TLS using the three transmon qubit devices on Chip-B. The parameters listed include TLS frequency, interaction strength g with the transmon qubit, as well as their T_1 relaxation times, with one standard deviation uncertainty quoted. The hosting transmon qubit device for the TLS and the specific cool-down cycle when these TLS were characterized, are specified at the end of the list. †: Measurements conducted using the direct TLS control method (Sec. III1), which likely results in shorter TLS T_1 measurements due to microwave generated quasiparticles. $ or -: Signify cases where the coupling strength g could not be extracted with high confidence. This might arise from small g values, or overlapping TLS avoided crossings, as identified in the SWAP spectroscopy. CD1/CD2: Indicate the cool-down cycle during which the TLS were measured.

	f_1 (GHz)	f_2 (GHz)	Median in/out (μs)	Mean in/out (μs)
Chip-A Q_1	-	5.796–5.798	948/2.3	835/31.2
Chip-A Q_2	4.975–5.653	5.690–6.274	1635/16.1	1635/16.1
Chip-A Q_3	4.510–4.692	5.487–5.735	524/5.6	1349/7.4
Chip-A Q_4	4.343–4.956	5.410–5.852	451/11.2	506/11.2
Chip-B Q_1	4.437–4.547	-	476/4.0	496/14.2
Chip-B Q_2	4.423–4.891	-	490/4.2	490/27.5
Chip-B Q_3	4.329–4.987	-	759/11.5	759/12.7
avg. BG	4.510–4.547	5.690–5.735	505/4.4	796/24.5
sim. BG	4.442	5.814	462/4.3	680/18.4

TABLE S5. **Acoustic bandgap and TLS T_1 enhancement on individual devices.** Here we outline the experimentally identified bandgap frequencies $[f_1, f_2]$ for individual transmon devices. These bandgaps are determined by minimizing the cost function in Eq. S4. Both the median and mean T_1 inside and outside the resultant bandgap are listed to demonstrate the robust two-orders-of-magnitude enhancement consistently observed across all devices. Additionally, the average bandgap determined using data from all TLS (avg. BG) is provided for reference. This average bandgap is compared to the prediction from COMSOL simulation (sim. BG), revealing a small difference of less than 100 MHz in their bandedge frequencies, which highlights the robustness of the fabrication process for the acoustic metamaterials.

	bound1 (GHz)	bound2 (GHz)
TLS1	0.41	-1.6
TLS3	0.59	-4.4
TLS4	0.65	-1.3
TLS5	0.87	-4.1

TABLE S6. **Bounds on TLS anharmonicity from SWAP spectroscopy of two excitations.** The values bound1 and bound2 are determined by the experiments shown in Fig. S19, assuming positive and negative anharmonicity, that satisfy $\alpha > \text{bound1} > 0$ and $\alpha < \text{bound2} < 0$, respectively.

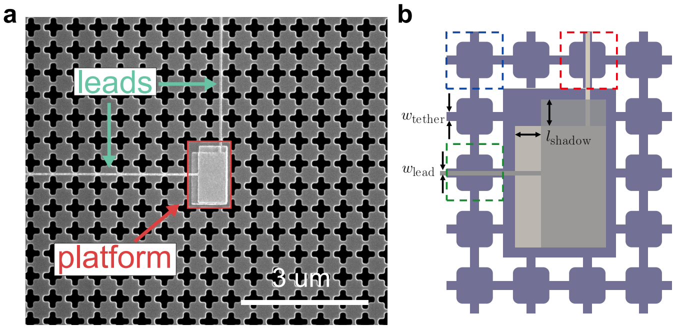

FIG. S1. **JJ embedded in the acoustic metamaterial. a,** SEM showcasing the JJ, the rectangular platform, and the surrounding acoustic metamaterial. The JJ is positioned on top of an approximately $1 \times 1.6\ \mu m^2$ rectangular Si platform, highlighted in red. The JJ is formed by two Al layers sandwiching a thin AlO_x barrier layer. Each Al layer has a narrow and long lead, indicated by turquoise arrows. These JJ leads pass through the acoustic metamaterials and form a SQUID loop. **b,** Schematic topview of **a**. For our fabricated devices, we have $w_{\text{lead}} = 45$ nm, $w_{\text{tether}} = 72$ nm, and $l_{\text{shadow}} \approx 150$ nm. The alignment between the JJ and the Si tether is better than ~ 10 nm. Based on our device geometry, the unit cell of the acoustic structures has three different types, showcased in the blue, red, and green dashed boxes, representing the Si cross-shield, Si cross-shield with 30 nm thick JJ lead, and Si cross-shield with 50 nm thick JJ lead, respectively.

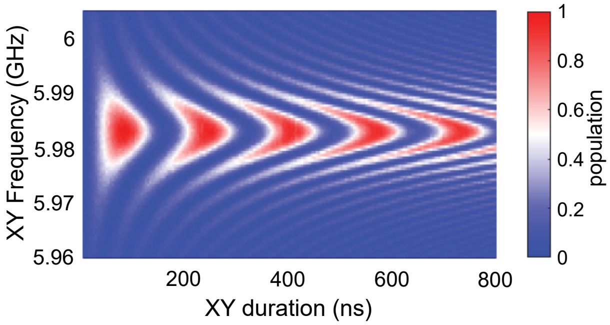

FIG. S2. **Rabi chevron of a representative hybrid transmon qubit device.** The measurements presented correspond to Q_4 of Chip-A, conducted during the first cool down.

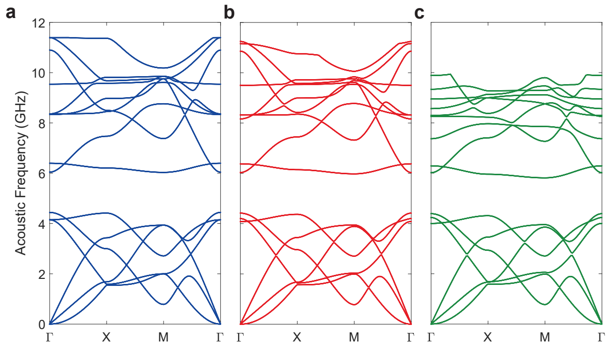

FIG. S3. **Simulated acoustic band structures of the cross-shield acoustic metamaterial unit cell.** Utilizing COMSOL simulations, we present the acoustic band structures of the three types of unit cells: **a,** silicon only cross-shield, **b,** silicon cross-shield with 30nm thick Al lead passing through, and **c,** silicon cross-shield with 50nm thick Al lead passing through. The Si cross-shield geometry remains the same for all three cases, with Si device layer thickness of 220 nm. The simulated bandgap frequencies are listed in Table S2.

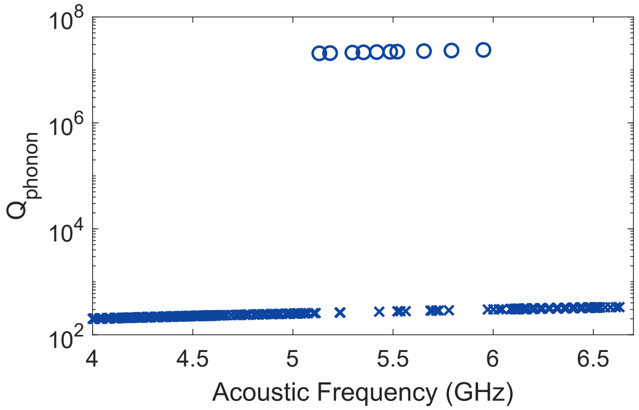

FIG. S4. **Simulated acoustic phonon modes supported by the Si platform and Josephson junction structure on top.** The platform is surrounded by the acoustic bandgap metamaterials. Perfectly Matched Layer (PML) boundary condition is used outside the metamaterial structure. The high-Q modes inside the bandgap are denoted by circles. The low-Q modes inside the bandgap and the alteration in the bandgap are attributed to edge effects due to limited simulation size and coarse meshing under the computational resource constraints.

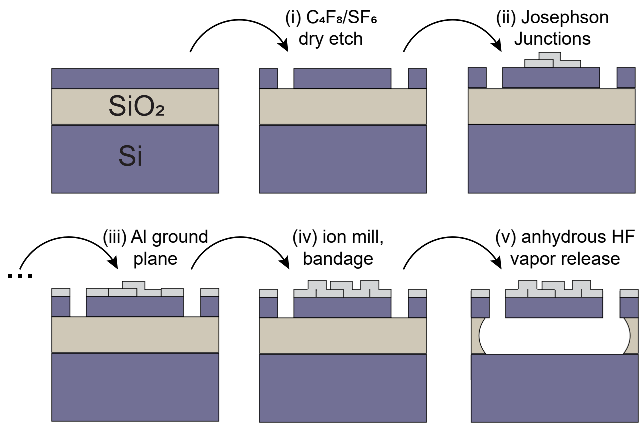

FIG. S5. **Fabrication process of the hybrid device on SOI substrate.** All beam writes employ 100 keV electron-beam lithography (Raith EBPG 5200). All metal depositions are realized by electron beam evaporation (Plassys MEB550S) and a liftoff process.

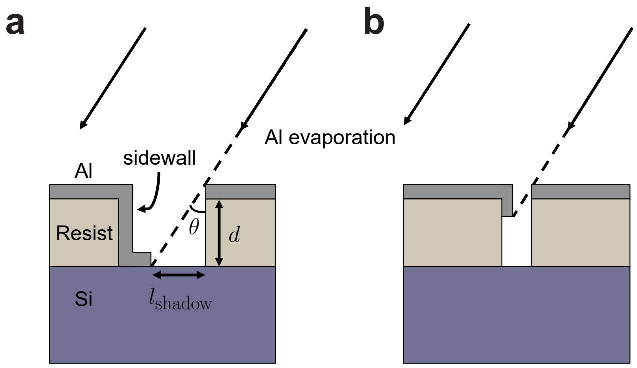

FIG. S6. **Sideview of angled evaporation in a single layer resist process. a,** From a geometric standpoint, there is a shadow area of size $l_{\text{shadow}} = d \tan\theta$ without metalization, where d is the thickness of the resist, and θ the evaporation angle from normal incidence. In addition, during angled evaporation of Al, due to the lack of an undercut in a single layer resist process, Al is deposited both on the Si substrate (excluding the shadow area) and on the sidewalls of the resist. The latter sometimes remains as free-standing vertical sidewalls post the liftoff process. Notably, these residual vertical sidewalls do not influence the transmon qubit performance in our experiment. **b,** In scenarios where the feature size is smaller than the shadow size, e.g., $w_{\text{lead}} \ll l_{\text{shadow}}$, there is no metalization on Si. Consequently, a Manhattan-style JJ that takes advantage of the shadow could avoid the formation of parasitic junctions within target regions.

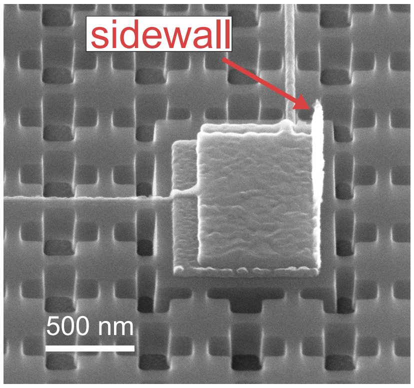

FIG. S7. **Angled SEM of a Josephson junction with Al sidewall**. The free-standing Al sidewall comes from the liftoff process, which is a consequence of the lack of an undercut in conjugation with angled evaporation in our single resist JJ process.

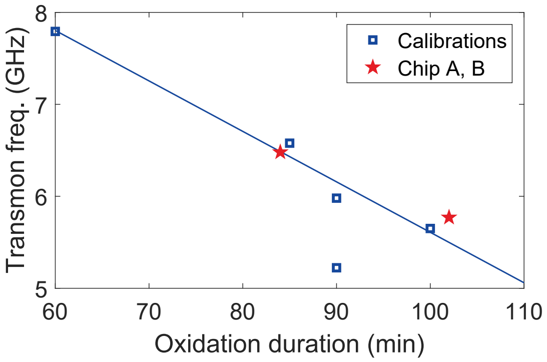

FIG. S8. **Transmon frequency-dependence on the JJ oxidation time.** All the collected data points except for one exhibit an empirical linear relation, which we use to inform the fabrication of Chip-A and Chip-B. The one outlier data point (90 min, 5.22 GHz) represent a chip that was aged for roughly a month before measurements were taken, which possibly explains the abnormal behavior. The pentagrams represent frequencies of Q_1's of Chip-A and Chip-B, which agree well with the empirical linear fit.

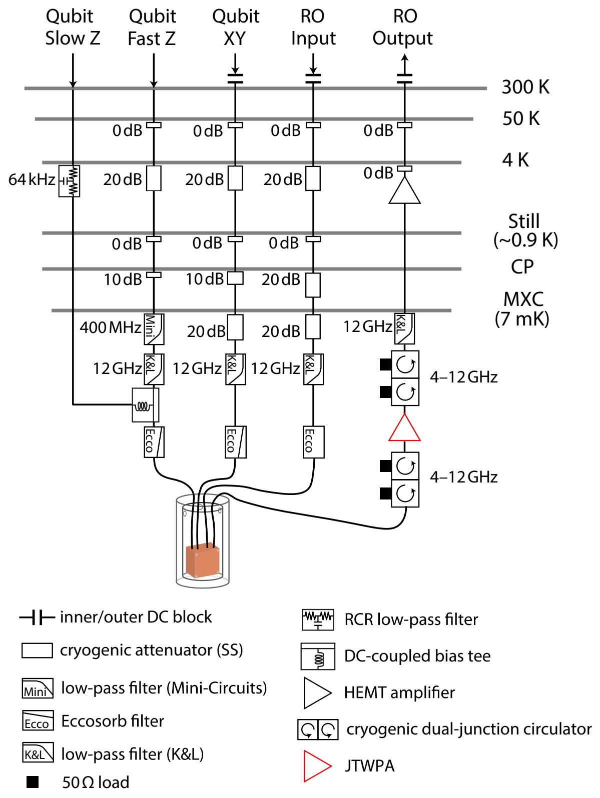

FIG. S9. **Schematic of the measurement setup inside the dilution fridge.** The setup includes slow and fast Z lines for qubit frequency tuning, XY lines for qubit drive, and RO input, RO output lines for qubit dispersive readout. The values for cryogenic attenuation and filters at different temperature stages are listed in the diagram. The pump line for JTWPA is not shown in the diagram for brevity.

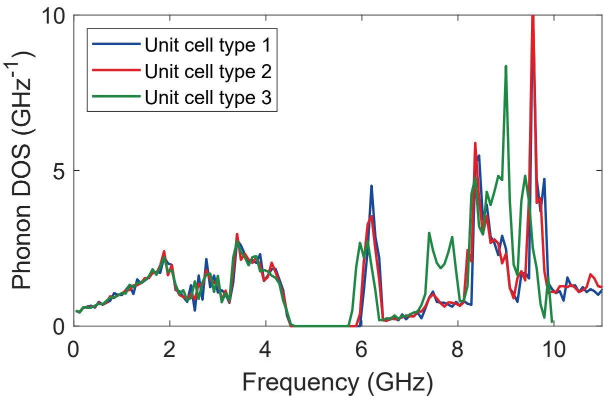

FIG. S10. **Simulated phonon density of states for the three unit cell types.** Colors in the plot correspond to the three types of unit cells as illustrated in the blue, red, and green dashed boxes in Fig. S1b.

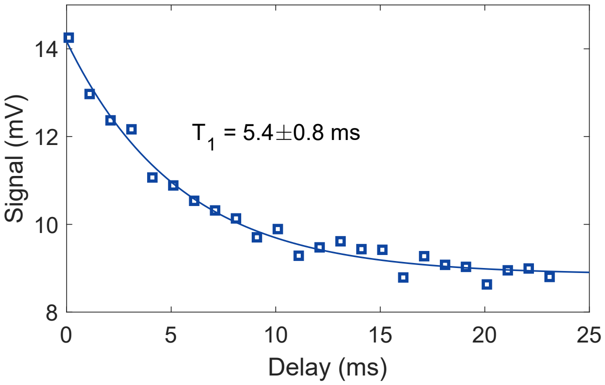

FIG. S11. T_1 **relaxation curve of TLS22.** This measurement represents the longest T_1 relaxation time of all the 56 TLS characterized. Markers represent experimental data, and the solid line is a simple exponential fit, given by $A\exp(-t/T_1)+B$. Note the 'delay' represented on the x-axis is in units of 'millisecond', as opposed to 'microsecond' used in other plots, to accommodate the long relaxation time.

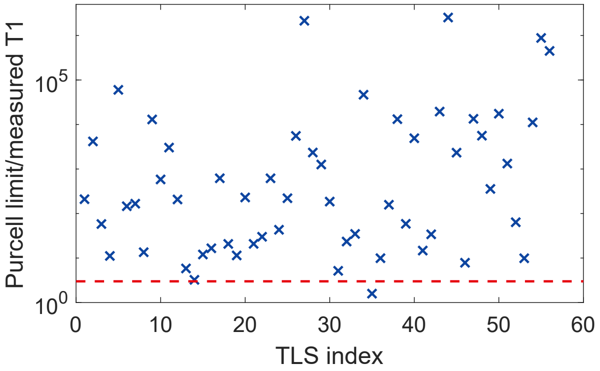

FIG. S12. **Purcell limit due to the transmon qubit.** Blue crosses represent the ratio between the estimated Purcell limit for each TLS due to the transmon qubit, and their measured T_1 lifetime. The red dashed line is a guide for the eye, at a value of 3. Except for TLS35 (which has therefore been discarded in further TLS analysis), the Purcell limit is at least a factor of 3× larger than the measured TLS T_1, strongly suggesting the transmon is not limiting the measured T_1 relaxation time of TLS.

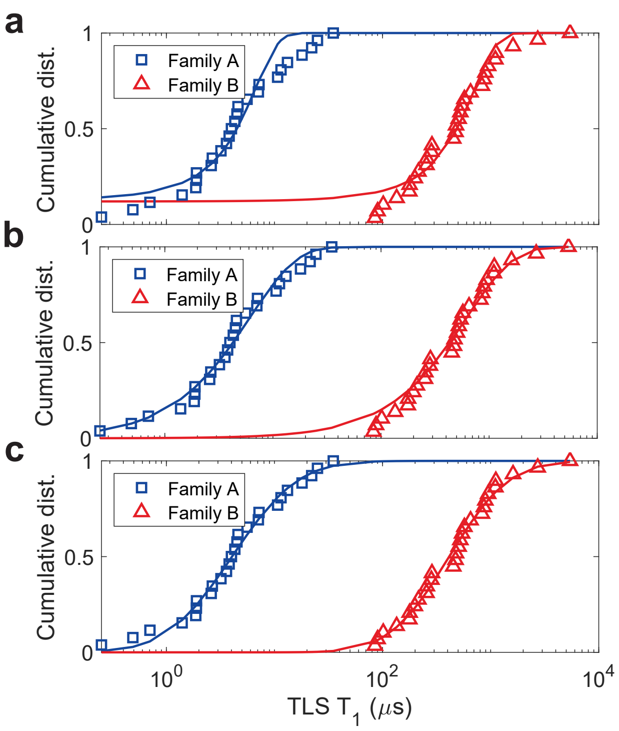

FIG. S13. **Cumulative distributions of TLS T_1 values**. TLS T_1 distributions for family A (blue squares) and family B (red triangles) are fitted to three commonly used models: **a,** the normal distribution, **b,** the exponential distribution, and **c,** the log-normal distribution. The solid lines in each subfigure correspond to the fits using the respective models. The log-normal distribution in **c** emerges as the best overall fit to the data. This distribution model yields median T_1 values of 4.1 ± 0.2 μs, skewness of 8.3 for Family A and 414 ± 17 μs, skewness of 6.2 for Family B.

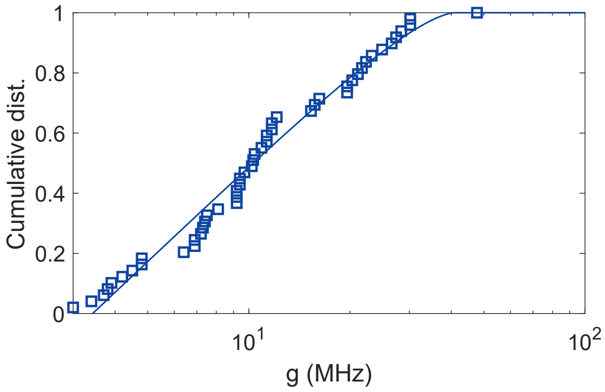

FIG. S14. **TLS cumulative distribution over coupling strength** g. Blue square markers represent experimental data, and blue solid line is fitting to the STM prediction of Eq. S3, which yields $\sigma = 0.6$ GHz$^{-1}\mu$m^{-2}.

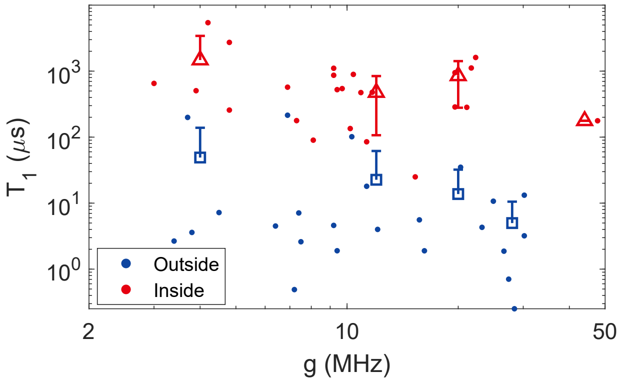

FIG. S15. **Scatter plot of TLS T_1 vs coupling strength g.** Blue and red filled circles represent the T_1 values for TLS outside and inside the average acoustic bandgap (avg. BG), respectively. The corresponding mean T_1 values (binned every 8 MHz by coupling strength g) are depicted in blue open squares and red open triangles. Errorbars denote one standard deviation. Missing lower part of errorbars marks standard deviation larger or equal to the mean value.

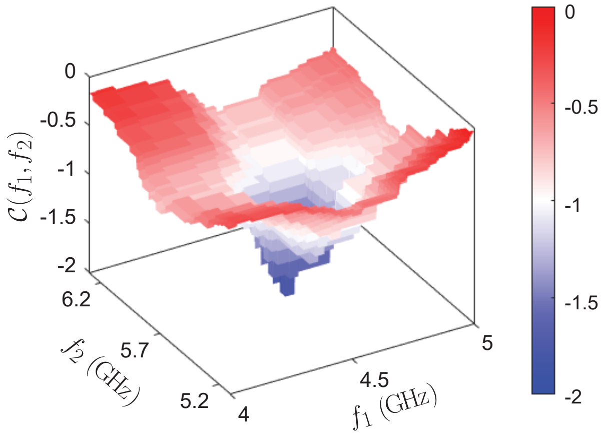

FIG. S16. **Landscape of the cost function $\mathcal{C}(f_1, f_2)$.** $\mathcal{C}_{\min} = -1.98$ identifies the average acoustic bandgap across all seven transmon devices, with $f_{1,\text{avg.bg}} \in [4.510, 4.547]$ GHz for the lower bandedge and $f_{2,\text{avg.bg}} \in [5.690, 5.735]$ GHz for the upper bandedge.

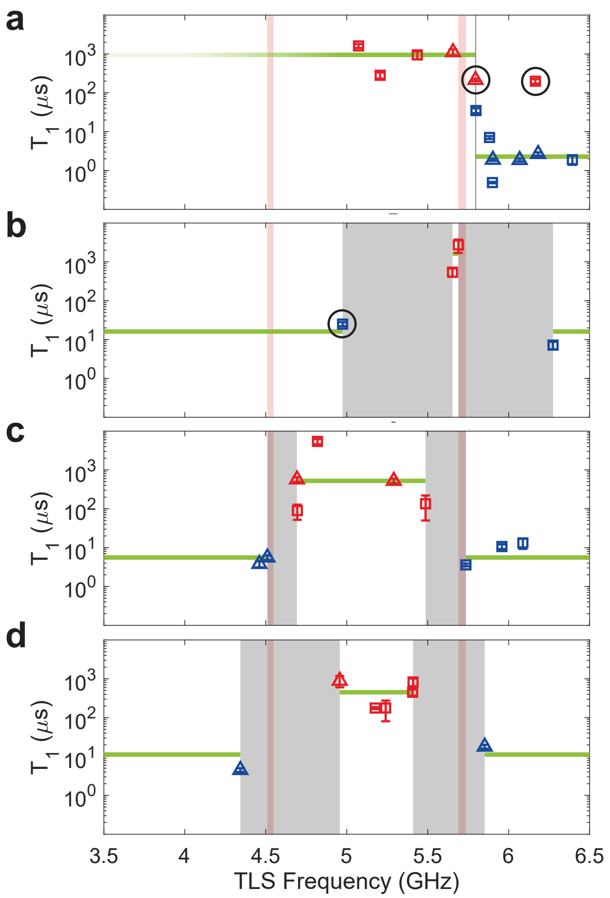

FIG. S17. **TLS T_1 measured on individual transmon devices on Chip-A. a,** Chip-A Q_1, **b,** Chip-A Q_2, **c,** Chip-A Q_3, and **d,** Chip-A Q_4. Blue and red markers denote TLS belonging to family A and family B, respectively. Additionally, square and triangular markers differentiate between TLS characterized during the first and second cool-down cycles. The gray shading represents the frequencies of the bandedges determined using the cost function for each individual device, while the pink shading corresponds to the average bandgap, serving as a reference. Solid green lines are guides to the eye, illustrating median TLS T_1 values both inside and outside the acoustic bandgap. Outlier TLS, classified using the average acoustic bandgaps, are marked by black circles. Data in **a, c, d** shows no apparent correlation between the TLS measured during different cool-downs. In the cases of **a** and **b**, upward shifts in bandgap frequencies are observed, which are attributed to fabrication disorder.

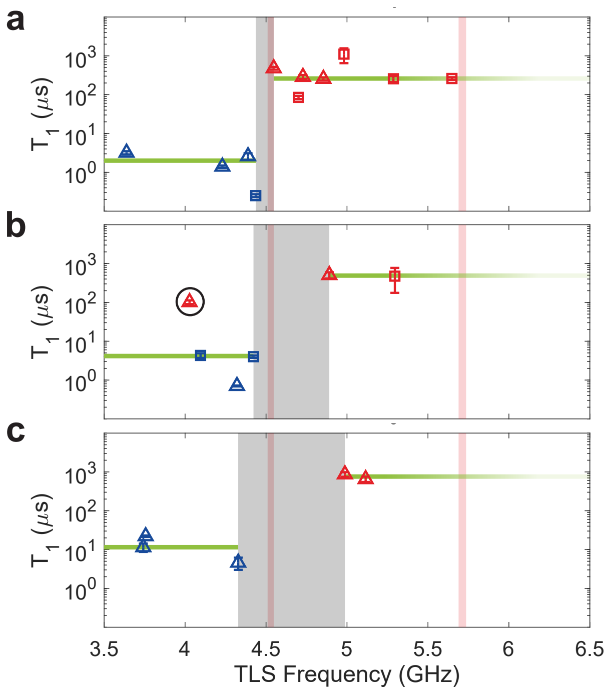

FIG. S18. **TLS T_1 measured on individual transmon devices on Chip-B. a,** Chip-B Q_1, **b,** Chip-B Q_2, and **c,** Chip-B Q_3. Blue and red markers denote TLS belonging to family A and family B, respectively. Additionally, square and triangular markers differentiate between TLS characterized during the first and second cool-down cycles. The gray shading represents the frequencies of the bandedges determined using the cost function for each individual device, while the pink shading corresponds to the average bandgap, serving as a reference. Solid green lines are guides to the eye, illustrating median TLS T_1 values both inside and outside the acoustic bandgap. Outlier TLS, classified using the average acoustic bandgaps, are marked by black circles. Data in **a, b** shows no apparent correlation between the TLS measured during different cool-downs.

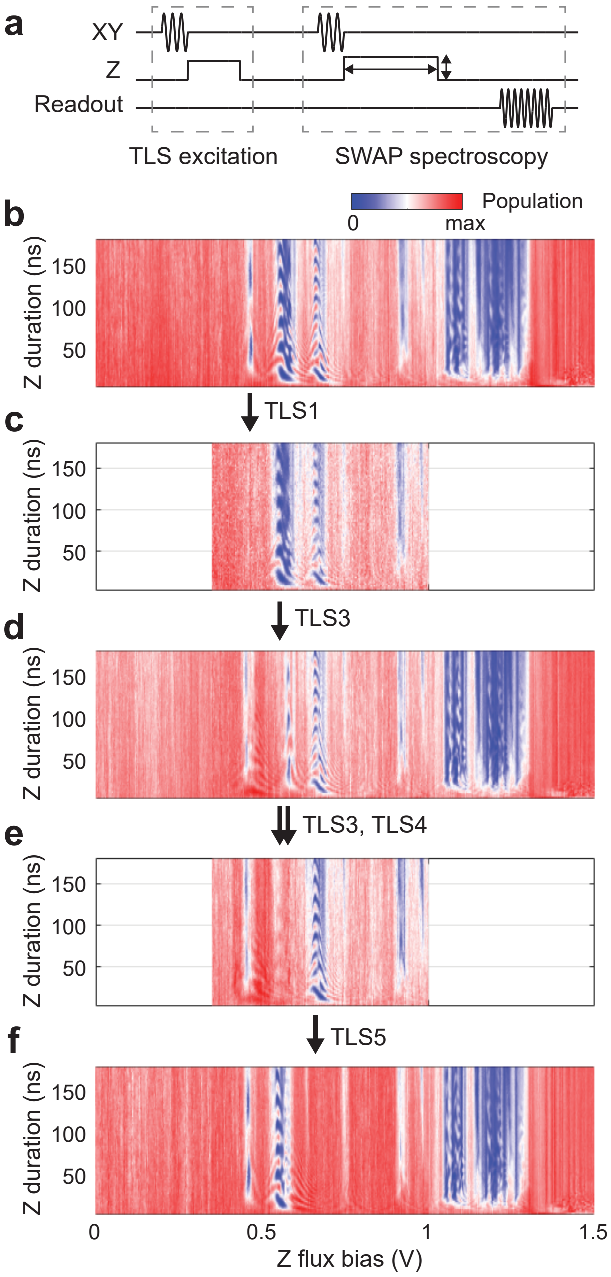

FIG. S19. **SWAP spectroscopy of two quanta of excitations. a,** Experimental sequence for the two excitation SWAP spectroscopy experiment, involving the initial excitation of the target TLS, followed by a regular SWAP spectroscopy that exchanges a second excitation from the transmon qubit. **b,** Reference SWAP spectroscopy when all TLS are in their ground states $|0\rangle$. **c,** SWAP spectroscopy with TLS1 in the excited-state $|1\rangle$. **d,** SWAP spectroscopy with TLS3 in excited-state $|1\rangle$. **e,** SWAP spectroscopy with both TLS3, TLS4 in excited-state $|1\rangle$. **f,** SWAP spectroscopy with TLS5 is in excited-state $|1\rangle$. Notably, TLS3 and TLS5 have stronger coupling $g \sim 20$ MHz, making it easier to resolve the presence of vacuum Rabi oscillations at lower frequencies. As a result, we selected TLS3 and TLS5 for a larger range scan of up to 1.5 V flux bias.

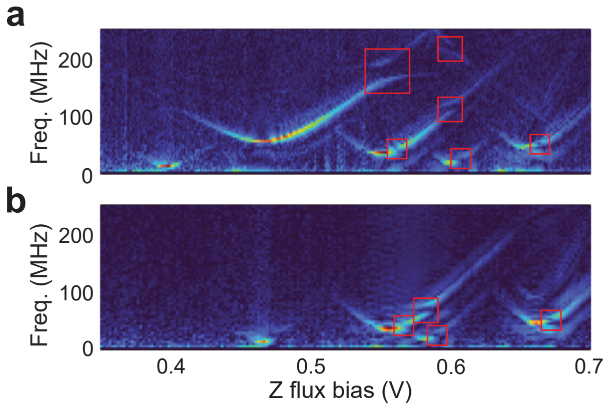

FIG. S20. **Fourier transform of SWAP spectroscopy: a,** of Fig. 2d of main text, and **b,** of Fig. S19b. The two datasets are taken from the same transmon device before and after a 200 mK thermal cycling. Red boxes highlight gaps in the vacuum Rabi frequencies, a result of TLS-transmon-TLS three-mode couplings.

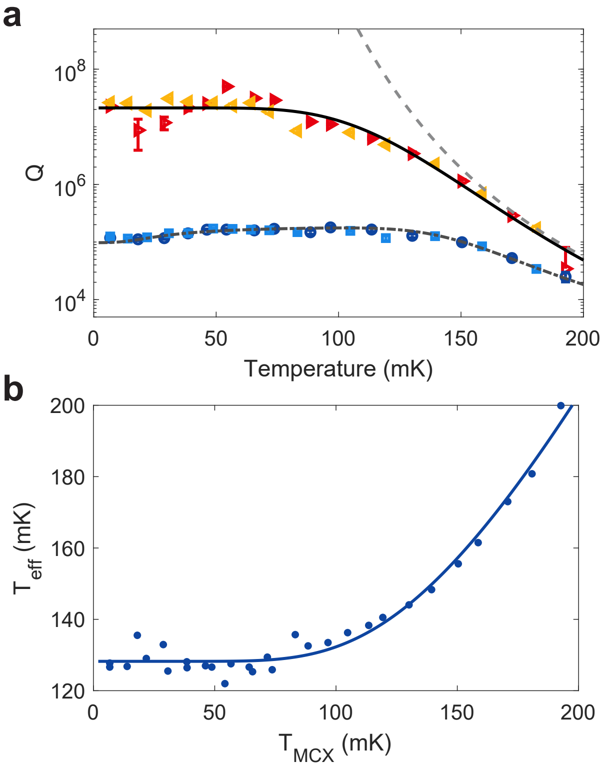

FIG. S21. **Investigating relaxation channels of TLS. a,** Plot of the Q-factor of both TLS5 and the transmon qubit as a function of the mixing plate temperature. The red (dark blue) markers denote measurements of the TLS (transmon qubit) during device warm-up (WU), and the yellow (light blue) markers during the device cooldown (CD). The gray dashed line corresponds to a phenomenological model of QP damping of TLS using the mixing plate temperature. The black solid line represents a correction to the gray dashed line, when using the effective temperature from **b**. The gray dash-dotted line is a fit to the transmon curve with a model including thermal saturation of weakly coupled TLS and damping from thermally-activated QPs, using the same effective temperature as TLS. **b,** Plot of the effective temperature against the mixing plate temperature. The effective temperature is deduced by assuming a single TLS energy relaxation channel of QPs. The empirical fit assumes the functional form $T_{\rm eff} = A\sqrt{1 + B\tanh(C/T_{\rm MXC})}/\tanh(C/T_{\rm MXC})$.

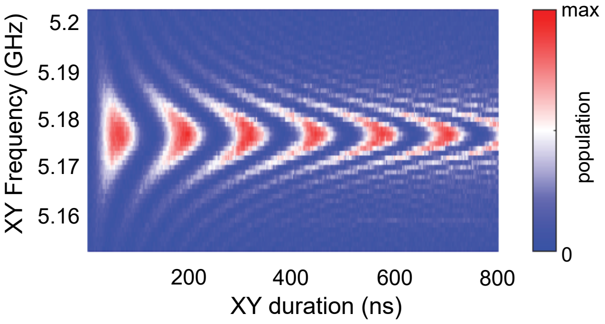

FIG. S22. **Rabi chevron of TLS31.** Measurements were taken on Q_4 of Chip-A during CD1. The interaction strength and detuning between the TLS and transmon qubit are $g = 47.7$ MHz, $\Delta = 1.7$ GHz. The XY driving power in this experiment is ~ 17 dB stronger than typically used for controlling the transmon qubit.

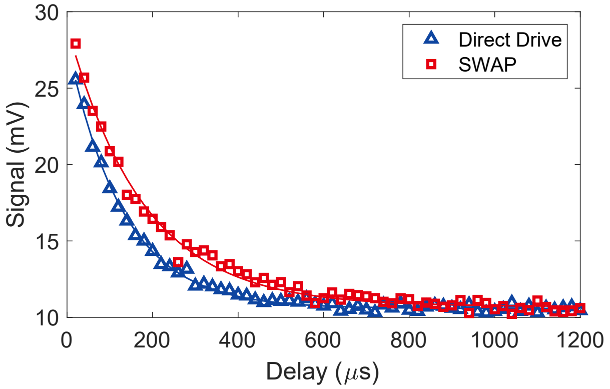

FIG. S23. T_1 **relaxation curves of TLS31**. We contrast T_1 measurements obtained using two different methods: preparing the initial TLS excited-state with a direct TLS π pulse (blue triangles) and through swapping the excitation from the transmon qubit (red squares). Exponential fits (solid lines) yield $T_1 = 129 \pm 5$ μs and $T_1 = 177 \pm 10$ μs for the direct drive and the SWAP methods, respectively.

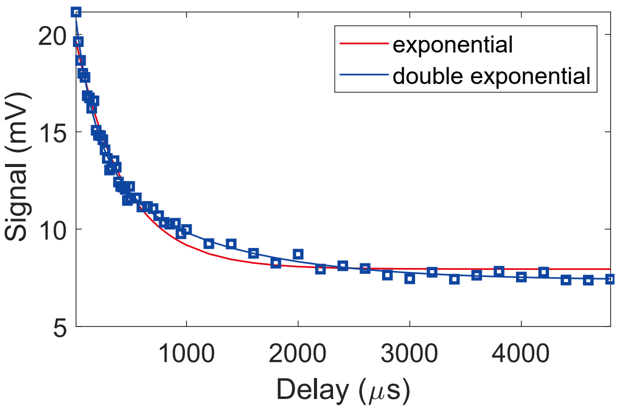

FIG. S24. T_1 **relaxation curve of TLS36.** We analyze the shape of the relaxation curve when the initial excited-state of TLS is prepared by direct XY drive. Fits using a simple exponential decay model (red solid line) and double exponential model (blue solid line) are superimposed. The data clearly demonstrates deviation from the simple exponential decay.

REFERENCES AND NOTES

1. W. A. Phillips, Tunneling states in amorphous solids. *J. Low Temp. Phys* **7**, 351–360 (1972).

2. P. W. Anderson, B. I. Halperin, C. M. Varma, Anomalous low-temperature thermal properties of glasses and spin glasses. *Philos. Mag. A J. Theor. Exp. Appl. Phys.* **25**, 1–9 (1972).

3. C. Müller, J. H. Cole, J. Lisenfeld, Towards understanding two-level-systems in amorphous solids: Insights from quantum circuits. *Rep. Prog. Phys.* **82**, 124501 (2019).

4. J. Gao, "The physics of superconducting microwave resonators," thesis, California Institute of Technology, Pasadena, CA (2008).

5. E. Paladino, Y. M. Galperin, G. Falci, B. L. Altshuler, 1/*f* noise: Implications for solid-state quantum information. *Rev. Mod. Phys.* **86**, 361–418 (2014).

6. C. Wang, C. Axline, Y. Y. Gao, T. Brecht, Y. Chu, L. Frunzio, M. H. Devoret, R. J. Schoelkopf, Surface participation and dielectric loss in superconducting qubits. *Appl. Phys. Lett.* **107**, 162601 (2015).

7. J. M. Gambetta, C. E. Murray, Y.-K.-K. Fung, D. T. McClure, O. Dial, W. Shanks, J. W. Sleight, M. Steffen, Investigating surface loss effects in superconducting transmon qubits. *IEEE Trans. Appl. Supercond.* **27**, 1700205 (2017).

8. P. V. Klimov, J. Kelly, Z. Chen, M. Neeley, A. Megrant, B. Burkett, R. Barends, K. Arya, B. Chiaro, Y. Chen, A. Dunsworth, A. Fowler, B. Foxen, C. Gidney, M. Giustina, R. Graff, T. Huang, E. Jeffrey, E. Lucero, J. Y. Mutus, O. Naaman, C. Neill, C. Quintana, P. Roushan, D. Sank, A. Vainsencher, J. Wenner, T. C. White, S. Boixo, R. Babbush, V. N. Smelyanskiy, H. Neven, J. M. Martinis, Fluctuations of energy-relaxation times in superconducting qubits. *Phys. Rev. Lett.* **121**, 090502 (2018).

9. M. Aspelmeyer, K. Schwab, Focus on mechanical systems at the quantum limit. *New J. Phys.* **10**, 095001 (2008).

10. E. A. Wollack, A. Y. Cleland, P. Arrangoiz-Arriola, T. P. McKenna, R. G. Gruenke, R. N. Patel, W. Jiang, C. J. Sarabalis, A. H. Safavi-Naeini, Loss channels affecting lithium niobate phononic crystal resonators at cryogenic temperature. *Appl. Phys. Lett.* **118**, 123501 (2021).

11. A. Y. Cleland, E. A. Wollack, A. H. Safavi-Naeini, Studying phonon coherence with a quantum sensor. *Nat. Commun.* **15**, 4979 (2024).

12. R. Rivière, S. Deléglise, S. Weis, E. Gavartin, O. Arcizet, A. Schliesser, T. J. Kippenberg, Optomechanical sideband cooling of a micromechanical oscillator close to the quantum ground state. *Phys. Rev. A* **83**, 063835 (2011).

13. G. S. MacCabe, H. Ren, J. Luo, J. D. Cohen, H. Zhou, A. Sipahigil, M. Mirhosseini, O. Painter, Nano-acoustic resonator with ultralong phonon lifetime. *Science* **370**, 840–843 (2020).

14. R. Barends, J. Kelly, A. Megrant, D. Sank, E. Jeffrey, Y. Chen, Y. Yin, B. Chiaro, J. Mutus, C. Neill, P. O'Malley, P. Roushan, J. Wenner, T. C. White, A. N. Cleland, J. M. Martinis, Coherent josephson qubit suitable for scalable quantum integrated circuits. *Phys. Rev. Lett.* **111**, 080502 (2013).

15. J. J. Burnett, A. Bengtsson, M. Scigliuzzo, D. Niepce, M. Kudra, P. Delsing, J. Bylander, Decoherence benchmarking of superconducting qubits. *npj Quantum Inf.* **5**, 54 (2019).

16. S. Schlör, J. Lisenfeld, C. Müller, A. Bilmes, A. Schneider, D. P. Pappas, A. V. Ustinov, M. Weides, Correlating decoherence in transmon qubits: Low frequency noise by single fluctuators. *Phys. Rev. Lett.* **123**, 190502 (2019).

17. P. Krantz, M. Kjaergaard, F. Yan, T. P. Orlando, S. Gustavsson, W. D. Oliver, A quantum engineer's guide to superconducting qubits. *Appl. Phys. Rev.* **6**, 021318 (2019).

18. M. Kjaergaard, M. E. Schwartz, J. Braumüller, P. Krantz, J. I.-J. Wang, S. Gustavsson, W. D. Oliver, Superconducting qubits: Current state of play. *Annu. Rev. Condens. Matter Phys.* **11**, 369–395 (2020).

19. J. Lisenfeld, A. Bilmes, A. V. Ustinov, Enhancing the coherence of superconducting quantum bits with electric fields. *npj Quantum Inf.* **9**, 8 (2023).

20. C. Müller, A. Shnirman, Y. Makhlin, Relaxation of josephson qubits due to strong coupling to two-level systems. *Phys. Rev. B* **80**, 134517 (2009).

21. K. Agarwal, I. Martin, M. D. Lukin, E. Demler, Polaronic model of two-level systems in amorphous solids. *Phys. Rev. B* **87**, 144201 (2013).

22. R. O. Behunin, F. Intravaia, P. T. Rakich, Dimensional transformation of defect-induced noise, dissipation, nonlinearity. *Phys. Rev. B* **93**, 224110 (2016).

23. Y. J. Rosen, M. A. Horsley, S. E. Harrison, E. T. Holland, A. S. Chang, T. Bond, J. L. DuBois, Protecting superconducting qubits from phonon mediated decay. *Appl. Phys. Lett.* **114**, 202601 (2019).

24. J. Lisenfeld, A. Bilmes, A. Megrant, R. Barends, J. Kelly, P. Klimov, G. Weiss, J. M. Martinis, A. V. Ustinov, Electric field spectroscopy of material defects in transmon qubits. *npj Quantum Inf.* **5**, 105 (2019).

25. M. Spiecker, P. Paluch, N. Gosling, N. Drucker, S. Matityahu, D. Gusenkova, S. Günzler, D. Rieger, I. Takmakov, F. Valenti, P. Winkel, R. Gebauer, O. Sander, G. Catelani, A. Shnirman, A. V. Ustinov, W. Wernsdorfer, Y. Cohen, I. M. Pop, Two-level system hyperpolarization using a quantum szilard engine. *Nat. Phys.* **19**, 1320–1325 (2023).

26. H. Paik, D. I. Schuster, L. S. Bishop, G. Kirchmair, G. Catelani, A. P. Sears, B. R. Johnson, M. J. Reagor, L. Frunzio, L. I. Glazman, S. M. Girvin, M. H. Devoret, R. J. Schoelkopf, Observation of high coherence in josephson junction qubits measured in a three-dimensional circuit qed architecture. *Phys. Rev. Lett.* **107**, 240501 (2011).

27. A. Bruno, G. de Lange, S. Asaad, K. L. van der Enden, N. K. Langford, L. DiCarlo, Reducing intrinsic loss in superconducting resonators by surface treatment and deep etching of silicon substrates. *Appl. Phys. Lett.* **106**, 182601 (2015).

28. S. Oh, K. Cicak, J. S. Kline, M. A. Sillanpää, K. D. Osborn, J. D. Whittaker, R. W. Simmonds, D. P. Pappas, Elimination of two level fluctuators in superconducting quantum bits by an epitaxial tunnel barrier. *Phys. Rev. B* **74**, 100502 (2006).

29. J. B. Chang, M. R. Vissers, A. D. Córcoles, M. Sandberg, J. Gao, D. W. Abraham, J. M. Chow, J. M. Gambetta, M. Beth Rothwell, G. A. Keefe, M. Steffen, D. P. Pappas, Improved superconducting qubit coherence using titanium nitride. *Appl. Phys. Lett.* **103**, 012602 (2013).

30. A. P. M. Place, L. V. H. Rodgers, P. Mundada, B. M. Smitham, M. Fitzpatrick, Z. Leng, A. Premkumar, J. Bryon, A. Vrajitoarea, S. Sussman, G. Cheng, T. Madhavan, H. K. Babla, X. H. Le, Y. Gang, B. Jäck, A. Gyenis, N. Yao, R. J. Cava, N. P. de Leon, A. A. Houck, New material platform for superconducting transmon qubits with coherence times exceeding 0.3 milliseconds. *Nat. Commun.* **12**, 1779 (2021).

31. J. Koch, T. M. Yu, J. Gambetta, A. A. Houck, D. I. Schuster, J. Majer, A. Blais, M. H. Devoret, S. M. Girvin, R. J. Schoelkopf, Charge-insensitive qubit design derived from the cooper pair box. *Phys. Rev. A* **76**, 042319 (2007).

32. J. A. Schreier, A. A. Houck, J. Koch, D. I. Schuster, B. R. Johnson, J. M. Chow, J. M. Gambetta, J. Majer, L. Frunzio, M. H. Devoret, S. M. Girvin, R. J. Schoelkopf, Suppressing charge noise decoherence in superconducting charge qubits. *Phys. Rev. B* **77**, 180502 (2008).

33. A. J. Keller, P. B. Dieterle, M. Fang, B. Berger, J. M. Fink, O. Painter, Al transmon qubits on silicon-on-insulator for quantum device integration. *Appl. Phys. Lett.* **111**, 042603 (2017).

34. J. Chan, A. H. Safavi-Naeini, J. T. Hill, S. Meenehan, O. Painter, Optimized optomechanical crystal cavity with acoustic radiation shield. *Appl. Phys. Lett.* **101**, 081115 (2012).

35. R. Zhao, S. Park, T. Zhao, M. Bal, C. McRae, J. Long, D. Pappas, Merged-element transmon. *Phys. Rev. Appl.* **14**, 064006 (2020).

36. H. Mamin, E. Huang, S. Carnevale, C. Rettner, N. Arellano, M. Sherwood, C. Kurter, B. Trimm, M. Sandberg, R. Shelby, M. Mueed, B. Madon, A. Pushp, M. Steffen, D. Rugar, Merged-element transmons: Design and qubit performance. *Phys. Rev. Appl.* **16**, 024023 (2021).

37. J. M. Martinis, K. B. Cooper, R. McDermott, M. Steffen, M. Ansmann, K. D. Osborn, K. Cicak, S. Oh, D. P. Pappas, R. W. Simmonds, C. C. Yu, Decoherence in josephson qubits from dielectric loss. *Phys. Rev. Lett.* **95**, 210503 (2005).

38. M. Neeley, M. Ansmann, R. C. Bialczak, M. Hofheinz, N. Katz, E. Lucero, A. O'Connell, H. Wang, A. N. Cleland, J. M. Martinis, Process tomography of quantum memory in a josephson-phase qubit coupled to a two-level state. *Nat. Phys.* **4**, 523–526 (2008).

39. Y. Shalibo, Y. Rofe, D. Shwa, F. Zeides, M. Neeley, J. M. Martinis, N. Katz, Lifetime and coherence of two-level defects in a josephson junction. *Phys. Rev. Lett.* **105**, 177001 (2010).

40. B.-J. Liu, Y.-Y. Wang, T. Sheffer, C. Wang, Observation of discrete charge states of a coherent two-level system in a superconducting qubit. arXiv:2401.12183 [quant-ph] (2024).

41. J. Lisenfeld, C. Müller, J. H. Cole, P. Bushev, A. Lukashenko, A. Shnirman, A. V. Ustinov, Measuring the temperature dependence of individual two-level systems by direct coherent control. *Phys. Rev. Lett.* **105**, 230504 (2010).

42. J. Lisenfeld, A. Bilmes, S. Matityahu, S. Zanker, M. Marthaler, M. Schechter, G. Schön, A. Shnirman, G. Weiss and A. V. Ustinov, Decoherence spectroscopy with individual two-level tunneling defects. *Sci. Rep.* **6**, 23786 (2016).

43. M. H. Abobeih, J. Cramer, M. A. Bakker, N. Kalb, D. J. Twitchen, M. Markham, T. H. Taminiau, One-second coherence for a single electron spin coupled to a multi-qubit nuclear-spin environment. *Nat. Commun.* **9**, 2552 (2018).

44. G. Catelani, J. Koch, L. Frunzio, R. J. Schoelkopf, M. H. Devoret, L. I. Glazman, Quasiparticle relaxation of superconducting qubits in the presence of flux. *Phys. Rev. Lett.* **106**, 077002 (2011).

45. K. D. Crowley, R. A. McLellan, A. Dutta, N. Shumiya, A. P. M. Place, X. H. Le, Y. Gang, T. Madhavan, N. Khedkar, Y. C. Feng, E. A. Umbarkar, X. Gui, L. V. H. Rodgers, Y. Jia, M. M. Feldman, S. A. Lyon, M. Liu, R. J. Cava, A. A. Houck, N. P. de Leon, Disentangling losses in tantalum superconducting circuits. *Phys. Rev. X* **13**, 041005 (2023).

46. T.-S. Kê, Experimental evidence of the viscous behavior of grain boundaries in metals. *Phys. Rev.* **71**, 533–546 (1947).

47. A. P. Vepsäläinen, A. H. Karamlou, J. L. Orrell, A. S. Dogra, B. Loer, F. Vasconcelos, D. K. Kim, A. J. Melville, B. M. Niedzielski, J. L. Yoder, S. Gustavsson, J. A. Formaggio, B. A. VanDevender, W. D. Oliver, Impact of ionizing radiation on superconducting qubit coherence. *Nature* **584**, 551–556 (2020).

48. S. Gustavsson, F. Yan, G. Catelani, J. Bylander, A. Kamal, J. Birenbaum, D. Hover, D. Rosenberg, G. Samach, A. P. Sears, S. J. Weber, J. L. Yoder, J. Clarke, A. J. Kerman, F. Yoshihara, Y. Nakamura, T. P. Orlando, W. D. Oliver, Suppressing relaxation in superconducting qubits by quasiparticle pumping. *Science* **354**, 1573–1577 (2016).

49. K. Serniak, M. Hays, G. de Lange, S. Diamond, S. Shankar, L. D. Burkhart, L. Frunzio, M. Houzet, M. H. Devoret, Hot nonequilibrium quasiparticles in transmon qubits. *Phys. Rev. Lett.* **121**, 157701 (2018).

50. A. Bilmes, S. Zanker, A. Heimes, M. Marthaler, G. Schön, G. Weiss, A. V. Ustinov, J. Lisenfeld, Electronic decoherence of two-level systems in a josephson junction. *Phys. Rev. B* **96**, 064504 (2017).

51. T. Connolly, P. D. Kurilovich, S. Diamond, H. Nho, C. G. L. Bøttcher, L. I. Glazman, V. Fatemi, M. H. Devoret, Coexistence of nonequilibrium density and equilibrium energy distribution of quasiparticles in a superconducting qubit. *Phys. Rev. Lett.* **132**, 217001 (2024).

52. S. E. de Graaf, S. Un, A. G. Shard, T. Lindström, Chemical and structural identification of material defects in superconducting quantum circuits. *Mater. Quantum. Technol.* **2**, 032001 (2022).

53. T. H. Taminiau, J. J. T. Wagenaar, T. van der Sar, F. Jelezko, V. V. Dobrovitski, R. Hanson, Detection and control of individual nuclear spins using a weakly coupled electron spin. *Phys. Rev. Lett.* **109**, 137602 (2012).

54. C. L. Degen, F. Reinhard, P. Cappellaro, Quantum sensing. *Rev. Mod. Phys.* **89**, 035002 (2017).

55. L. Faoro and L. B. Ioffe, Interacting tunneling model for two-level systems in amorphous materials and its predictions for their dephasing and noise in superconducting microresonators. *Phys. Rev. B* **91**, 014201 (2015).

56. S. M. Meißner, A. Seiler, J. Lisenfeld, A. V. Ustinov, G. Weiss, Probing individual tunneling fluctuators with coherently controlled tunneling systems. *Phys. Rev. B* **97**, 180505 (2018).

57. P. Dutta and P. M. Horn, Low-frequency fluctuations in solids:1/*f* noise. *Rev. Mod. Phys.* **53**, 497–516 (1981).

58. I. Siddiqi, Engineering high-coherence superconducting qubits. *Nat. Rev. Mater.* **6**, 875–891 (2021).

59. N. P. de Leon, K. M. Itoh, D. Kim, K. K. Mehta, T. E. Northup, H. Paik, B. S. Palmer, N. Samarth, S. Sangtawesin, D. W. Steuerman, Materials challenges and opportunities for quantum computing hardware. *Science* **372**, eabb2823 (2021).

60. C. E. Murray, Material matters in superconducting qubits. *Mater. Sci. Eng. R Rep.* **146**, 100646 (2021).

61. A. M. Zagoskin, S. Ashhab, J. R. Johansson, F. Nori, Quantum two-level systems in josephson junctions as naturally formed qubits. *Phys. Rev. Lett.* **97**, 077001 (2006).

62. M. Chen, W. K. C. Sun, K. Saha, J.-C. Jaskula, P. Cappellaro, Protecting solid-state spins from a strongly coupled environment. *New J. Phys.* **20**, 063011 (2018).

63. M. Odeh, K. Godeneli, E. Li, R. Tangirala, H. Zhou, X. Zhang, Z.-H. Zhang, A. Sipahigil, Non-markovian dynamics of a superconducting qubit in a phononic bandgap. arXiv:2312.01031 [quant-ph] (2023).

64. M. McEwen, K. C. Miao, J. Atalaya, A. Bilmes, A. Crook, J. Bovaird, J. M. Kreikebaum, N. Zobrist, E. Jeffrey, B. Ying, A. Bengtsson, H.-S. Chang, A. Dunsworth, J. Kelly, Y. Zhang, E. Forati, R. Acharya, J. Iveland, W. Liu, S. Kim, B. Burkett, A. Megrant, Y. Chen, C. Neill, D. Sank, M. Devoret, A. Opremcak, Resisting high-energy impact events through gap engineering in superconducting qubit arrays. arXiv:2402.15644 [quant-ph] (2024).

65. F. Yoshihara, K. Harrabi, A. O. Niskanen, Y. Nakamura, J. S. Tsai, Decoherence of flux qubits due to 1/f flux noise. *Phys. Rev. Lett.* **97**, 167001 (2006).

66. K. Kakuyanagi, T. Meno, S. Saito, H. Nakano, K. Semba, H. Takayanagi, F. Deppe, A. Shnirman, Dephasing of a superconducting flux qubit. *Phys. Rev. Lett.* **98**, 047004 (2007).

67. R. C. Bialczak, R. McDermott, M. Ansmann, M. Hofheinz, N. Katz, E. Lucero, M. Neeley, A. D. O'Connell, H. Wang, A. N. Cleland, J. M. Martinis, 1/f flux noise in josephson phase qubits. *Phys. Rev. Lett.* **99**, 187006 (2007).

68. D. A. Rower, L. Ateshian, L. H. Li, M. Hays, D. Bluvstein, L. Ding, B. Kannan, A. Almanakly, J. Braumüller, D. K. Kim, A. Melville, B. M. Niedzielski, M. E. Schwartz, J. L. Yoder, T. P. Orlando, J. I.-J. Wang, S. Gustavsson, J. A. Grover, K. Serniak, R. Comin, W. D. Oliver, Evolution of $1/f$ flux noise in superconducting qubits with weak magnetic fields. *Phys. Rev. Lett.* **130**, 220602 (2023).

69. B. L. Dwyer, L. V. Rodgers, E. K. Urbach, D. Bluvstein, S. Sangtawesin, H. Zhou, Y. Nassab, M. Fitzpatrick, Z. Yuan, K. De Greve, E. L. Peterson, H. Knowles, T. Sumarac, J.-P. Chou, A. Gali, V. Dobrovitski, M. D. Lukin, N. P. de Leon, Probing spin dynamics on diamond surfaces using a single quantum sensor. *PRX Quantum* **3**, 040328 (2022).

70. M. Klotz, K. G. Fehler, R. Waltrich, E. S. Steiger, S. Häußler, P. Reddy, L. F. Kulikova, V. A. Davydov, V. N. Agafonov, M. W. Doherty, A. Kubanek, Prolonged orbital relaxation by locally modified phonon density of states for the siv^{-} center in nanodiamonds. *Phys. Rev. Lett.* **128**, 153602 (2022).

71. K. Kuruma, B. Pingault, C. Chia, M. Haas, G. D. Joe, D. R. Assumpcao, S. W. Ding, C. Jin, C. J. Xin, M. Yeh, N. Sinclair, M. Loncar, Engineering phonon-qubit interactions using phononic crystals. arXiv:2310.06236 [quant-ph] (2023).

72. K. Zhang, M.-M. Li, Q. Liu, H.-F. Yu, Y. Yu, Bridge-free fabrication process for Al/AlO_x /Al Josephson junctions. *Chin. Phys. B* **26**, 078501 (2017).

73. V. S. Ferreira, G. Kim, A. Butler, H. Pichler, O. Painter, Deterministic generation of multidimensional photonic cluster states with a single quantum emitter. *Nat. Phys.* **20**, 865–870 (2024).

74. X. Zhang, E. Kim, D. K. Mark, S. Choi, O. Painter, A superconducting quantum simulator based on a photonic-bandgap metamaterial. *Science* **379**, 278–283 (2023).

75. M. A. Rol, L. Ciorciaro, F. K. Malinowski, B. M. Tarasinski, R. E. Sagastizabal, C. C. Bultink, Y. Salathe, N. Haandbaek, J. Sedivy, L. DiCarlo, Time-domain characterization and correction of on-chip distortion of control pulses in a quantum processor. *Appl. Phys. Lett.* **116**, 054001 (2020).

76. C. Wang, Y. Y. Gao, I. M. Pop, U. Vool, C. Axline, T. Brecht, R. W. Heeres, L. Frunzio, M. H. Devoret, G. Catelani, L. I. Glazman, R. J. Schoelkopf, Measurement and control of quasiparticle dynamics in a superconducting qubit. *Nat. Commun.* **5**, 5836 (2014).

77. U. Vool, I. M. Pop, K. Sliwa, B. Abdo, C. Wang, T. Brecht, Y. Y. Gao, S. Shankar, M. Hatridge, G. Catelani, M. Mirrahimi, L. Frunzio, R. J. Schoelkopf, L. I. Glazman, M. H. Devoret, Non-poissonian quantum jumps of a fluxonium qubit due to quasiparticle excitations. *Phys. Rev. Lett.* **113**, 247001 (2014).

78. A. Lupaşcu, P. Bertet, E. F. C. Driessen, C. J. P. M. Harmans, J. E. Mooij, One- and two-photon spectroscopy of a flux qubit coupled to a microscopic defect. *Phys. Rev. B* **80**, 172506 (2009).

79. P. Bushev, C. Müller, J. Lisenfeld, J. H. Cole, A. Lukashenko, A. Shnirman, A. V. Ustinov, Multiphoton spectroscopy of a hybrid quantum system. *Phys. Rev. B* **82**, 134530 (2010).

80. J. H. Cole, C. Müller, P. Bushev, G. J. Grabovskij, J. Lisenfeld, A. Lukashenko, A. V. Ustinov, A. Shnirman, Quantitative evaluation of defect-models in superconducting phase qubits. *Appl. Phys. Lett.* **97**, 252501 (2010).

81. G. J. Grabovskij, P. Bushev, J. H. Cole, C. Müller, J. Lisenfeld, A. Lukashenko, A. V. Ustinov, Entangling microscopic defects via a macroscopic quantum shuttle. *New J. Phys.* **13**, 063015 (2011).

82. M. Lucas, A. V. Danilov, L. V. Levitin, A. Jayaraman, A. J. Casey, L. Faoro, A. Y. Tzalenchuk, S. E. Kubatkin, J. Saunders, S. E. de Graaf, Quantum bath suppression in a superconducting circuit by immersion cooling. *Nat. Commun.* **14**, 3522 (2023).

83. C. D. Wilen, S. Abdullah, N. A. Kurinsky, C. Stanford, L. Cardani, G. D'Imperio, C. Tomei, L. Faoro, L. B. Ioffe, C. H. Liu, A. Opremcak, B. G. Christensen, J. L. DuBois, R. McDermott, Correlated charge noise and relaxation errors in superconducting qubits. *Nature* **594**, 369–373 (2021).

84. M. McEwen, L. Faoro, K. Arya, A. Dunsworth, T. Huang, S. Kim, B. Burkett, A. Fowler, F. Arute, J. C. Bardin, A. Bengtsson, A. Bilmes, B. B. Buckley, N. Bushnell, Z. Chen, R. Collins, S. Demura, A. R. Derk, C. Erickson, M. Giustina, S. D. Harrington, S. Hong, E. Jeffrey, J. Kelly, P. V. Klimov, F. Kostritsa, P. Laptev, A. Locharla, X. Mi, K. C. Miao, S. Montazeri, J. Mutus, O. Naaman, M. Neeley, C.

Neill, A. Opremcak, C. Quintana, N. Redd, P. Roushan, D. Sank, K. J. Satzinger, V. Shvarts, T. White, Z. J. Yao, P. Yeh, J. Yoo, Y. Chen, V. Smelyanskiy, J. M. Martinis, H. Neven, A. Megrant, L. Ioffe, R. Barends, Resolving catastrophic error bursts from cosmic rays in large arrays of superconducting qubits. *Nat. Phys.* **18**, 107–111 (2022).

85. T. Thorbeck, A. Eddins, I. Lauer, D. T. McClure, M. Carroll, Two-level-system dynamics in a superconducting qubit due to background ionizing radiation. *PRX Quantum.* **4**, 020356 (2023).

86. J. Burnett, L. Faoro, I. Wisby, V. L. Gurtovoi, A. V. Chernykh, G. M. Mikhailov, V. A. Tulin, R. Shaikhaidarov, V. Antonov, P. J. Meeson, A. Y. Tzalenchuk, T. Lindstroem, Evidence for interacting two-level systems from the 1/*f* noise of a superconducting resonator. *Nat. Commun.* **5**, 4119 (2014).

87. G. Zolfagharkhani, A. Gaidarzhy, S.-B. Shim, R. L. Badzey, P. Mohanty, Quantum friction in nanomechanical oscillators at millikelvin temperatures. *Phys. Rev. B.* **72**, 224101 (2005).

88. R. . Orbach and B. Bleaney, Spin-lattice relaxation in rare-earth salts. *Proc. R. Soc. Lond. A Math. Phys. Sci.* **264**, 458–484 (1961).

89. M. C. Cambria, A. Gardill, Y. Li, A. Norambuena, J. R. Maze, S. Kolkowitz, State-dependent phonon-limited spin relaxation of nitrogen-vacancy centers. *Phys. Rev. Res.* **3**, 013123 (2021).

90. G. P. Srivastava, *The Physics of Phonons* (Routledge, 1990).

91. G. Catelani, R. J. Schoelkopf, M. H. Devoret, L. I. Glazman, Relaxation and frequency shifts induced by quasiparticles in superconducting qubits. *Phys. Rev. B* **84**, 064517 (2011).

92. G. S. Paraoanu, Microwave-induced coupling of superconducting qubits. *Phys. Rev. B* **74**, 140504 (2006).

93. C. Rigetti and M. Devoret, Fully microwave-tunable universal gates in superconducting qubits with linear couplings and fixed transition frequencies. *Phys. Rev. B* **81**, 134507 (2010).

94. M. Chen, M. Hirose, P. Cappellaro, Measurement of transverse hyperfine interaction by forbidden transitions. *Phys. Rev. B* **92**, 020101 (2015).

95. I. M. Pop, K. Geerlings, G. Catelani, R. J. Schoelkopf, L. I. Glazman, M. H. Devoret, Coherent suppression of electromagnetic dissipation due to superconducting quasiparticles. *Nature* **508**, 369–372 (2014).
